# Complete mitogenome sequences of four flatfishes (Pleuronectiformes) reveal a novel gene arrangement of L-strand coding genes

**DOI:** 10.1186/1471-2148-13-173

**Published:** 2013-08-20

**Authors:** Wei Shi, Xiao-Li Dong, Zhong-Ming Wang, Xian-Guang Miao, Shu-Ying Wang, Xiao-Yu Kong

**Affiliations:** 1 CAS Key Laboratory of Tropical Marine Bio-resources and Ecology, South China Sea Institute of Oceanology, Chinese Academy of Sciences, 164 West Xingang Road, Guangzhou 510301, PR China; 2 Heilongjiang River Fisheries Research Institute, Chinese Academy of Fishery Science, 232 Hesong Street, Harbin 150070, PR China; 3 Key Laboratory of Sustainable Utilization of Technology Research for Fishery Resource of Zhejiang Province, Marine Fisheries Research Institute of Zhejiang, Zhoushan 316100, China

## Abstract

**Background:**

Few mitochondrial gene rearrangements are found in vertebrates and large-scale changes in these genomes occur even less frequently. It is difficult, therefore, to propose a mechanism to account for observed changes in mitogenome structure. Mitochondrial gene rearrangements are usually explained by the recombination model or tandem duplication and random loss model.

**Results:**

In this study, the complete mitochondrial genomes of four flatfishes, *Crossorhombus azureus* (blue flounder), *Grammatobothus krempfi, Pleuronichthys cornutus*, and *Platichthys stellatus* were determined. A striking finding is that eight genes in the *C. azureus* mitogenome are located in a novel position, differing from that of available vertebrate mitogenomes. Specifically, the *ND6* and seven tRNA genes (the *Q, A, C, Y, S*_*1*_*, E, P* genes) encoded by the L-strand have been translocated to a position between *tRNA-T* and *tRNA-F* though the original order of the genes is maintained.

**Conclusions:**

These special features are used to suggest a mechanism for *C. azureus* mitogenome rearrangement. First, a dimeric molecule was formed by two monomers linked head-to-tail, then one of the two sets of promoters lost function and the genes controlled by the disabled promoters became pseudogenes, non-coding sequences, and even were lost from the genome. This study provides a new gene-rearrangement model that accounts for the events of gene-rearrangement in a vertebrate mitogenome.

## Background

Mitochondrial DNA (mtDNA) of vertebrate is a circular DNA molecule of 15–20 kb normally containing 13 protein-coding genes, 22 tRNA genes, two rRNA genes, one origin of replication on the light-strand (O_L_), and a single control region (CR). The CR is essential for the initiation of transcription and for replication of the heavy strand [1]. Most genes are encoded by the heavy (H-) strand; only the *ND6* gene and eight tRNA genes are encoded by the light (L-) strand. Transcription of L- or H- strand occurs from the light-strand promoter (LSP) or heavy-strand promoter (HSP) [2,3].

Currently, over 1700 complete mitochondrial genome (mitogenome) sequences from vertebrates are available, and although the gene order of most vertebrate mitogenomes is conserved, mtDNA gene rearrangements have been found in some groups [4-7]. Thus far, three models have been used to explain gene rearrangements in animal mtDNA. First, the recombination model, initially proposed for gene rearrangements in nuclear genomes, is characterized by breakage and rejoining of participating DNA strands [8]. This model has been adopted to account for changes in mitochondrial gene order in frog, bird, mussels, and others [5,9,10]. Another commonly accepted hypothesis is the tandem duplication and random loss (TDRL) model, which posits that rearrangements of mitochondrial gene order have occurred via tandem duplications of some genes followed by random deletion of some of the duplications [11,12]. This model is widely used to explain gene rearrangements in vertebrate mtDNA [4,7,13,14]. Lavrov et al. [15] created a model of tandem duplication and non-random loss (TDNL) to explain the gene rearrangements in two millipede mtDNA genomes (*Narceus annularus* and *Thyropygus sp.*). According to this model, the mitogenome duplicates to form a dimer genome (two monomer-mitogenomes linked head-to-tail). The duplication is then followed by gene loss determined by transcriptional polarity rather than via random gene loss [15]. Since then, this model has been used to explain the formation of only a few gene rearrangements all in invertebrate mitogenomes [16-18]. To date, no vertebrate mtDNA arrangements have been fit to the Lavrov et al. [15] model.

Here we describe the complete mitogenomes of four flatfishes, *Crossorhombus azureus* (blue flounder), *Grammatobothus krempfi*, *Pleuronichthys cornutus*, and *Platichthys stellatus*, all of which belong to the superfamily Pleuronectoidea. *C. azureaus* and *G. krempfi* are members of the Bothidae family, while the other two fishes are in the Pleuronectidae family. The gene order of the *G. krempfi*, *P. cornutus* and *P. stellatus* mitogenomes is the same as that of a typical vertebrate. However, we have discovered a novel gene rearrangement in *C. azureus* mtDNA. From this mitogenome, a new model of gene rearrangement in the *C. azureus* lineage is inferred.

## Methods

### Sampling, DNA extraction, PCR and sequencing

Specimens of *C. azureus* (C. azu) were collected from Zhuhai of Guangdong province, *G. krempfi* (G. kre) from Xiangshan of Zhejiang province, *P. cornutus* (P. cor) and *P. stellatus* (P. ste) from Qingdao of Shandong province. A portion of the epaxial musculature was excised from fresh specimen and immediately stored at −70°C. Total genomic DNA was extracted using the SQ Tissue DNA Kit (OMEGA) following the manufacturer’s protocol. Based on alignments and comparisons of complete mitochondrial sequences of flatfishes, dozens of primer pairs were designed for amplification of the mtDNA genomes (Additional file 1: Table S1). More than 30 bp of overlapping fragments between tandem regions were used to ensure correct assembly and integrity of the complete sequence.

PCR was performed in a 25 μl reaction volume containing 2.0 mM MgCl_2_, 0.4 mM of each dNTP, 0.5 μM of each primer, 1.0 U of Taq polymerase (Takara, China), 2.5 μl of 10× Taq buffer, and approximately 50 ng of DNA template. PCR cycling conditions included an initial denaturation at 95°C for 3 min, 30–35 cycles at 94°C for 45 s, an annealing temperature of 45–55°C for 45 s, and elongation at 68–72°C for 1.5-5 min. The PCR reaction was completed by a final extension at 72°C for 5 min. The PCR products were purified with the Takara Agarose Gel DNA Purification Kit (Takara, China) and used directly as templates for cycle sequencing reactions. Sequence-specific primers were further designed and used as walking primers for both strands of each fragment with an ABI 3730 DNA sequencer (Applied Biosystems, USA). The sequences of the mtDNAs of *C. azureus*, *G. krempfi*, *P. cornutus* and *P. stellatus* have been submitted to GenBank under the accession numbers JQ639068, JQ639069, JQ639071, NC_010966, respectively.

### Sequence analysis

Sequenced fragments were assembled to create complete mitochondrial genomes using CodonCode Aligner v3 and BioEdit v7 [19]. During the processing of large fragments and walking sequences, regular manual examinations were made to ensure reliable assembly of the genome sequence. Annotation and boundary determination of protein-coding and ribosomal RNA genes were performed using NCBI-BLAST (http://blast.ncbi.nlm.nih.gov/Blast.cgi). Transfer RNA genes and their secondary structures were identified using tRNAscan-SE 1.21 [20], setting the cut-off values to 1 when necessary. The gene maps of each of the four flatfish mitogenomes were generated using CGView [21]. Mitogenomes of eight other Pleuronectoidea fishes were retrieved from GenBank (Additional file 2: Table S2), including one Scophthalmidae specimen, *Scophthalmus maxima* (S. max); one Paralichthyidae fish, *Paralichthys olivaceus* (P. oli); and the other six Pleuronectidae fishes: *Kareius bicoloratus*, *Verasper variegatus* (V. var), *Verasper moseri* (V. mos), *Hippoglossus hippoglossus* (H. hip), *Hippoglossus stenolepis* (H. ste), and *Reinhardtius hippoglossoides* (R. hip).

## Results and discussion

**Figure 1:**
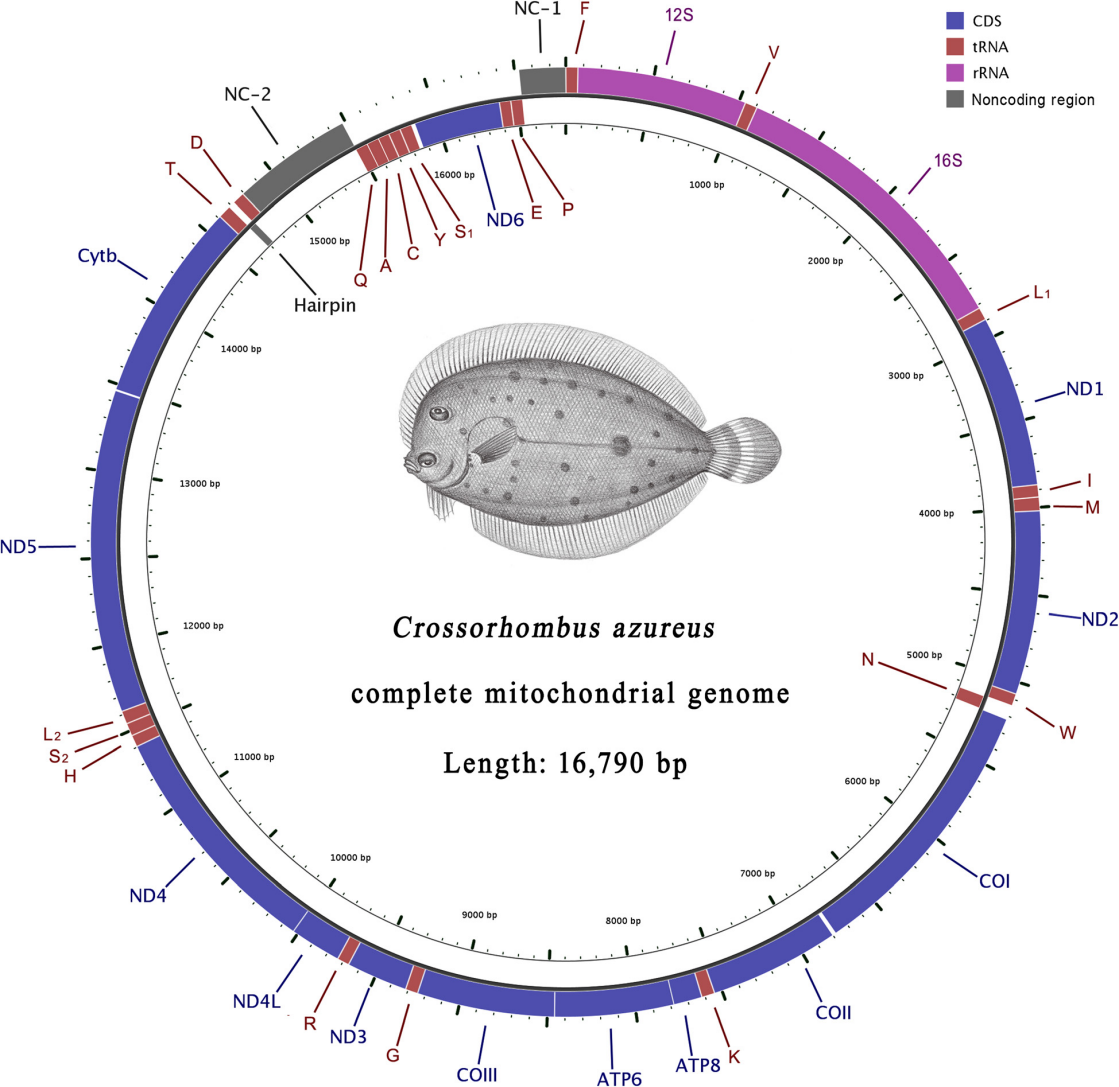
**Gene map of the mitochondrial genome of *C. azureus.***

The genomes of *C. azureus*, *G. krempfi*, *P. cornutus*, and *P. stellatus* are all circular molecules of 1,6790 bp, 1,6599 bp, 1,7469 and 1,7103 bp, respectively, and each contains 37 genes, as is typical for vertebrate mtDNAs (Figure 1, Additional file 3: Table S3 and Additional file 4: Figure S4).

### Novel gene order in the *C. azureus* mitogenome

**Figure 2:**
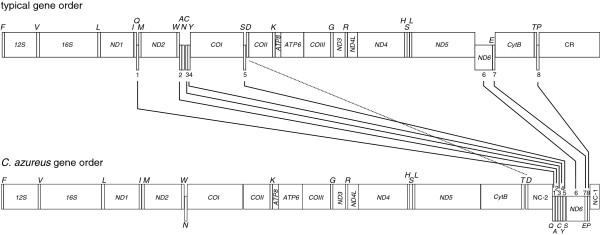
**Comparison of gene order between *C. azureus *and the typical fish mitogenome.** Arabic numerals indicate the relative order of rearranged genes on the L-strand: *Q-A-C-Y-S1-ND6-E-P*.

The arrangement of the 37 genes in *G. krempfi*, *P. cornutus* and *P. stellatus* mtDNA is identical to that of a typical vertebrate (Additional file 4: Figure S4). A striking finding in this study is that eight genes of the *C. azureus* mitogenome have a novel position differing from that of any other vertebrate mitogenome. In the blue flounder, the *ND6* and seven tRNA genes (the *Q, A, C, Y, S*_*1*_*, E, P* genes) encoded by the L-strand have been translocated to a position between *tRNA-T* and *tRNA-F*. Thus, with one exception, the genes with identical transcriptional polarities are clustered in the genome and separated by two non-coding regions. The exception is the L-strand-encoded *tRNA-N* gene located in a region with genes of the opposite transcriptional polarity (Figure 1). Interestingly, the original order of the rearranged genes, *Q-A-C-Y-S*_*1*_*-ND6-E-P*, is maintained (Figure 2). Analysis of 1750 vertebrate mitogenomes available in GenBank (as of Nov. 2012) revealed that none had a cluster of more than five genes encoded by the L-strand. Thus, the arrangement of genes in the blue flounder mitogenome appears to be unique in vertebrates. One additional translocation is noted: *tRNA-D* (encoded by H-strand) is translocated from its typical location between *COI* and *COII* to a position following *CytB* (Figure 2).

### CR variation in the *C. azureus* mitogenome

**Figure 3:**
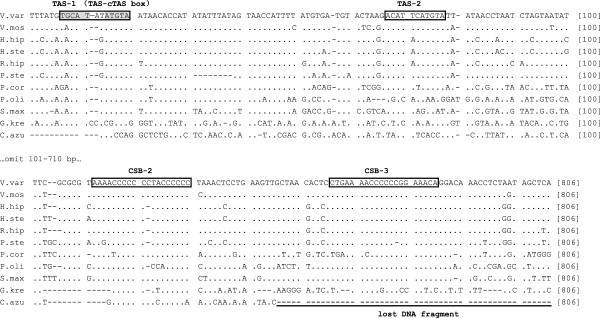
**Aligned CR sequences of ten Pleuronectoidea fish and the NC-2 sequence of *C. azureus*.** The boxed sequences indicate the Termination-Associated Sequences (TASs; Grayed sequences represent TAS-cTAS box) and Conserved Sequence Blocks (CSBs). The underlined block indicates the sequences of NC-2 lost in *C. azureus.* Abbreviation of fish names is given in Methods.

The CRs of *G. krempfi*, *P. cornutus,* and *P. stellatus* are located between *tRNA-P* and *tRNA-F*, as is typical, with lengths of 891 bp, 1,778 bp and 1,400 bp, respectively. Comparison of these CR sequences with those of seven other flatfishes reveals that the CR structure is typical for teleosts [22-25], including Termination-Associated Sequences (TAS-1, 2) and Conserved Sequence Blocks (CSB-2, 3). TAS-1 includes a typical TAS-complementary TAS block sequence (TAS-cTAS: TACAT-ATGTA) (Figure 3, Additional file 5: Figure S5). However, only a 263 bp non-coding fragment (NC-1) remains in the original CR location in the *C. azureus* mitogenome (Figure 1), and none of the TAS, CSB, or any other conserved sequences was observed. Another non-coding region of 687 bp (NC-2) was found between the *tRNA-D* and *tRNA-Q* genes, including possible TAS-1 and CSB-2 (Figures 1, 3, and Additional file 5: Figure S5). Accordingly, we consider NC-2 to be a part of the CR. However, CSB-3 and typical downstream sequences observed in other flatfish were not found (Figure 3, Additional file 5: Figure S5). Generally, the LSP and HSP are situated between the CSB and *tRNA-F*[1,3]. The lack of downstream sequences implies the loss of LSP and HSP in this partial CR.

### Location and sequence variations of O_L_ region in the *C. azureus* mitogenome

**Figure 4:**
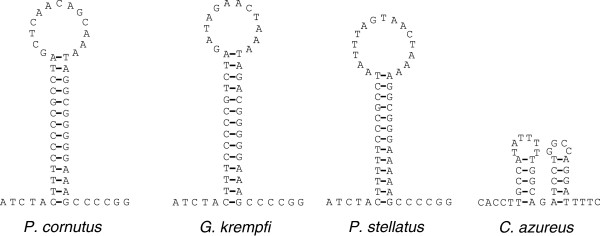
**Stem-loop structures of the O**_**L **_**in the *P. cornutus*, *G. krempfi*, and *P. stellatus *mitogenomes; and putative substitute of the O**_**L **_**for the *C. azureus *mitogenome*.***

The O_L_ sequences in *G. krempfi*, *P. cornutus*, and *P. stellatus* were found between *tRNA-N* and *tRNA-C* in the tRNA gene cluster known as the WANCY region (the tRNA cluster of tRNA-*Trp*, *Ala*, *Asn*, *Cys* and *Tyr*) as is typical for vertebrates [26-29]. These O_L_ sequences have the potential to fold into stable stem-loop structures with 13- or 14- bp stems and 13-, 14-, and 15-base loops (Figure 4). However, due to translocation of the *tRNA-A, C, and Y* genes in the *C. azureus* mitogenome*,* the WANCY region of this mitogenome contains only an 8-bp intergenic spacer between *tRNA-N* and *COI* genes, and is thus unable to form the stem-loop structure of the O_L_. O_L_ sequence loss has also been seen in some vertebrate mitogenomes, where it has been suggested that a sequence encoding a tRNA adopts a hairpin structure and acts as the O_L_[30-32].

### Gene rearrangement mechanism for the *C. azureus* mitogenome

Generally in vertebrate mitogenomes, small-scale gene rearrangements are rare and genomic-scale changes occur even less frequently [7], especially in teleostean fishes [28,33-35]. It is difficult, therefore, to propose a mechanism to account for the observed changes in genome structure. Gene rearrangement events are usually explained by the recombination or TDRL models [7]. The genes of the *C. azureus* mitogenome are extensively rearranged with clustering of eight of nine genes on the L-strand in the same polarity in an unchanged relative order. These special features provide a foundation on which to suggest a mechanism for gene-rearrangement in the *C. azureus* mitogenome. Though the gene rearrangement seen in *C. azureus* can be explained by recombination, TDRL or other models, using these models to explain observed *C. azureus* rearrangements is not as parsimonious as the model proposed below. For instance, to apply the recombination model to the *C. azureus* mitogenome, more than four recombination events would be required and each recombination event would need to translocate certain L-strand coding genes to the specific position at L-strand coding gene cluster. Since it is known that among the teleost fishes even single gene rearrangements caused by recombination are rare, this model seems an unlikely fit to the data. Similarly, using the tRNA mis-priming model [36] would require five or more specific tRNA mis-priming events. Lastly, apply tandem duplication “random loss” (TDRL) to the *C. azureus* mitogenome, the “loss” events, from the duplicated genome to the *C. azureus* type, shared very peculiar characteristic: only the L-strand coding gene including *ND6* and tRNA of *P*, *E*, *S*, *Y*, *C*, *A* and *Q* was translocated and grouped together. Instead, the rearrangement of the *C. azureus* genome including two groups of genes with different transcriptional polarities is better explained by the following model.

**Figure 5:**
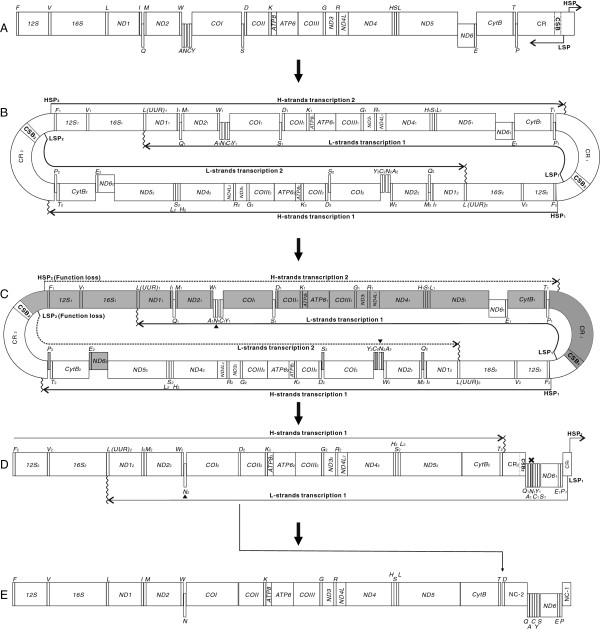
**Inferred intermediate steps from the ancestral gene order to that of the *C. azureus *mitogenome.** Protein-coding genes and CRs are indicated by boxes, and the tRNA genes are indicated by columns. Genes labeled above the diagram are encoded by the H-strand, those below the diagram by the L-strand. The LSP and HSP indicate the light-strand and heavy-strand promoters, respectively; CSB indicates Conserved Sequence Block. The direction of transcription is shown by arrows. The copied *tRNA-N* are marked by triangles. **(A)** ancestral gene order; **(B)** The dimeric molecule with two monomers linked head-to-tail; The locations of LSP_1, 2_, HSP_1, 2_ and *tRNA-L(UUR)*_*1, 2*_, 5’ end of CR indicate the proposed positions for transcription initiation and termination of the two monomers. **(C)** Functional loss of LSP_2_, HSP_2_; broken line indicates the disabled transcription regions; Dark gray box indicates the degeneration of LSP_2_, HSP_2_ and related genes. **(D)** Proposed translocation of *tRNA-D* is shown by arrow. **(E)** Gene order of the *C. azureus* mitogenome.

Because the gene order of 11 of 12 flatfish mitogenomes discussed in this paper (Additional file 2: Table S2) is the same as the typical arrangement, including one member of the Bothidae family, *G. krempfi*, we hypothesize that the ancestral mitochondrial gene arrangement in *C. azureus* (in the family Bothidae) was that of a typical vertebrate (Figure 5A). We further hypothesize that the processes leading to the observed blue flounder gene arrangement are as follows. The first step would have been a duplication of the entire mitogenome, resulting in a dimeric molecule with the two monomers linked head-to-tail (Figure 5B). The genes and CRs of the dimeric mtDNA are assumed to have retained their functions at this time, so that transcription could be initiated normally at the promoters (LSP_1_ and HSP_2_, LSP_2_ and HSP_1_) and transcription would be terminated at *tRNA-L (UUR)* for the L-strand and at part of the CR close to *tRNA-T* for the H-strand [37-39] (Figure 5B). Subsequently, the functionality of the promoters in one of the control regions (assumed to be LSP_2_ and HSP_2_) was lost or severely impaired due to mutation or fragment loss, thus the genes controlled by the disabled promoters (LSP_2_ and HSP_2_) would become pseudogenes (grayed regions, Figure 5C). These pseudogenes could then accumulate additional mutations to become shorter non-cording sequences or even be lost from the genome (Figure 5D). Consequently, the genes transcribed from LSP_1_ to *tRNA-L(UUR)*_*1*_ (gene block1: *P*_*1*_, *E*_*1*_, *ND6*_*1*_, *S*_*1*_, *Y*_*1*_, *C*_*1*_, *N*_*1*_, *A*_*1*_ and *Q*_*1*_) would be clustered together, and the other genes transcribed from HSP_1_ to part of the CR (gene block 2: *F*_*2*_, *12S*_*2*_*, V*_*2*_,……*ND5*_*2*_*CytB*_*2*_*, T*_*2*_) would also be clustered, with the exception of the retention of *tRNA-N*_*2*_ gene which clusters with genes of the opposite transcriptional polarity (Figure 5C,D).

The *tRNA-N* gene is located in WANCY region adjoining O_L_ and Seligmann and Krishnan [32] speculated that it not only was transcribed into tRNA-N, but also could form O_L_-like structures that may have functioned during mitochondrial replication of the L-strand. Therefore, although the *tRNA-N*_2_ should not be transcribed in the process shown in Figure 5C, it was still preserved because it functioned as O_L_ or assisted in O_L_ functioning during L-strand replication. In the following processes, due to degradation of *tRNA-L(UUR)*_*1*_ (the termination of L-strands transcription 1), transcription would be terminated at *tRNA-L(UUR)*_*2*_ instead of at *L(UUR)*_*1*_. Hence, the gene *tRNA-N*_*2*_ could be re-transcribed (Figure 5D). Finally, the *tRNA-N*_*2*_ gene was preserved while *N*_*1*_ was lost. Lastly, the gene *tRNA-D* was translocated from between *COI* and *COII* genes to a site between *tRNA-T* and CR. This event can be explained by tRNA mis-priming model or recombination event. Such translocations had been found in vertebrate and are relatively common in metazoan mitochondrial genome rearrangements [4,10,40]. Translocation of *tRNA-D* could have occurred either before or after the duplication and loss events postulated above. After the above rearrangements, a hybrid monomer-mitogenome (gene block1 and block2) would have been formed, in which genes with identical transcriptional polarity were placed into two clusters separated by two noncoding regions (Figure 5E).

### Details and support for the model

The inferred “dimer-mitogenome” intermediate of the *C. azureus* mtDNA (Figure 5B) could be formed by two entire mitogenomes or from two longer mtDNA fragments that include all L-coding genes (namely from *tRNA-Q* to CR, Figure 5A). While the duplication of a very large fragment is unusual in vertebrate mitogenomes, the dimeric mitogenome molecule has been observed in many animals [17,41,42] including almost all mammals [43]. Therefore, a duplication of the complete genome is more likely than the duplication of a very large fragment.

The inferred intermediate rearrangement for the *C. azureus* mitogenome is similar to that of the TDNL [15]. The crucial step in both models is that one set of light and heavy strand promoters lost function. The two non-coding regions (NC-1, NC-2) present in the *C. azureus* mitogenome provide evidence for this intermediate step. When comparing the CR structure with those of other fishes, we found that the 687 bp NC-2 region includes possible TAS-1 and CSB-2 sequences, but not the LSP or HSP (after CSB; Figure 3). This feature provides evidence that one set of transcriptional promoters in the CR lost function (Figure 5C). To date, no conserved sequences of the LSP and the HSP have been found in teleostean fishes. However, the logical position of the promoters in the *C*. *azureus* mitogenome would be in NC-1 for the following reasons. First, most researches [1,37,38] agrees that the HSP and LSP must be located very close to *tRNA-F* and the 5’ end of the 12S rRNA gene. NC-1 is the closest region to those genes. Second, NC-1 is located where the two gene clusters are separated by their transcription polarities, allowing transcription to originate in both directions (Figure 5D). According to previous studies, the LSP and HSP must be located in a non-coding region not far from 3’ end of CSB (close to the origin of replication for the H-strand: O_H_) because the RNA primer from LSP to O_H_ is necessary for mitochondrial replication [1,44]. Again, NC-1 is the closest, sufficiently long non-coding region located downstream of CSB (Figure 1, Additional file 3: Table S3a). In summary, the features of NC-1 support the interpretation that “the other CR retains the promoters” in our model.

## Conclusions

In summary, we determined the complete mitochondrial genomes of four flatfishes, *Crossorhombus azureus* (blue flounder), *Grammatobothus krempfi, Pleuronichthys cornutus*, and *Platichthys stellatus*. The genes of the *C. azureus* mitogenome are extensively rearranged with eight of nine genes on the L-strand in the same polarity and their relative order unchanged. A mechanism similar to the TDNL model is proposed to explain the origin of these special features. The model also explains the gene-rearrangements in which genes are clustered in the same polarity (L- or H-strand coding) with their relative order unchanged.

### Data accession

Sequences were deposited in the NCBI [JQ639068, JQ639069, JQ639071, NC_010966].

## Competing interests

The authors declare that they have no competing interests.

## Authors’ contributions

WS collected datasets, carried out experiments, and drafted the manuscript. XYK directed the whole research work and revised the manuscript. XLD, XGM, and SYW carried out partial experiments. All authors read and approved the final manuscript.

## Supplementary Material

Additional file 1: Table S1 The primers used for fragment amplification in four flatfish mitogenomes. Click here for file

Additional file 2: Table S2 Information of flatfishes used in this study. Click here for file

Additional file 3: Table S3 Organization of four flatfishes mitochondrial genomes. Click here for file

Additional file 4: Figure S4 Gene maps of the mitochondrial genome of *G. krempfi*, *P. cornutus* and *P. stellatus*. Click here for file

Additional file 5: Figure S5 Complete CR DNA fragments alignment of 11 Pleauonectoidea fishes. Click here for file

## References

[B1] Clayton DA Replication and transcription of vertebrate mitochondrial-DNA Annu Rev Cell Biol 1991 7 453 478 10.1146/annurev.cb.07.110191.002321 1809353

[B2] Shadel GS Clayton DA Mitochondrial DNA maintenance in vertebrates Annu Rev Biochem 1997 66 409 435 10.1146/annurev.biochem.66.1.409 9242913

[B3] Clayton DA Transcription and replication of mitochondrial DNA Hum Reprod 2000 15 Suppl 2 11 17 10.1093/humrep/15.suppl_2.11 11041509

[B4] Amer SA Kumazawa Y The mitochondrial genome of the lizard *Calotes versicolor*and a novel gene inversion in South Asian draconine agamids Mol Biol Evol 2007 24 6 1330 1339 10.1093/molbev/msm054 17379622

[B5] Sammler S Bleidorn C Tiedemann R Full mitochondrial genome sequences of two endemic Philippine hornbill species (Aves: Bucerotidae) provide evidence for pervasive mitochondrial DNA recombination BMC Genomics 2011 12 35 10.1186/1471-2164-12-35 21235758 PMC3025957

[B6] Zhou Y Zhang JY Zheng RQ Yu BG Yang G Complete nucleotide sequence and gene organization of the mitochondrial genome of *Paa spinosa* (Anura: Ranoidae) Gene 2009 447 2 86 96 10.1016/j.gene.2009.07.009 19631263

[B7] San Mauro D Gower DJ Zardoya R Wilkinson M A hotspot of gene order rearrangement by tandem duplication and random loss in the vertebrate mitochondrial genome Mol Biol Evol 2006 23 1 227 234 16177229 10.1093/molbev/msj025

[B8] Lunt DH Hyman BC Animal mitochondrial DNA recombination Nature 1997 387 6630 247 10.1038/387247a0 9153388

[B9] Ladoukakis ED Zouros E Recombination in animal mitochondrial DNA: evidence from published sequences Mol Biol Evol 2001 18 11 2127 2131 10.1093/oxfordjournals.molbev.a003755 11606710

[B10] Kurabayashi A Sumida M Yonekawa H Glaw F Vences M Hasegawa M Phylogeny, recombination, and mechanisms of stepwise mitochondrial genome reorganization in mantellid frogs from Madagascar Mol Biol Evol 2008 25 5 874 891 10.1093/molbev/msn031 18263605

[B11] Arndt A Smith MJ Mitochondrial gene rearrangement in the sea cucumber genus *Cucumaria* Mol Biol Evol 1998 15 8 1009 1016 10.1093/oxfordjournals.molbev.a025999 9718728

[B12] Moritz C Dowling TE Brown WM Evolution of animal mitochondrial-DNA - relevance for population biology and systematics Annu Rev Ecol Syst 1987 18 269 292 10.1146/annurev.es.18.110187.001413

[B13] Inoue JG Miya M Tsukamoto K Nishida M Evolution of the deep-sea gulper eel mitochondrial genomes: large-scale gene rearrangements originated within the eels Mol Biol Evol 2003 20 11 1917 1924 10.1093/molbev/msg206 12949142

[B14] Schirtzinger EE Tavares ES Gonzales LA Eberhard JR Miyaki CY Sanchez JJ Hernandez A Mueller H Graves GR Fleischer RC Multiple independent origins of mitochondrial control region duplications in the order Psittaciformes Mol Phylogenet Evol 2012 64 2 342 356 10.1016/j.ympev.2012.04.009 22543055 PMC4450661

[B15] Lavrov DV Boore JL Brown WM Complete mtDNA sequences of two millipedes suggest a new model for mitochondrial gene rearrangements: duplication and nonrandom loss Mol Biol Evol 2002 19 2 163 169 10.1093/oxfordjournals.molbev.a004068 11801744

[B16] Gai Y Song D Sun H Yang Q Zhou K The complete mitochondrial genome of *Symphylella sp.* (Myriapoda: Symphyla): extensive gene order rearrangement and evidence in favor of Progoneata Mol Phylogenet Evol 2008 49 2 574 585 10.1016/j.ympev.2008.08.010 18782622

[B17] Beckenbach AT Mitochondrial genome sequences of Nematocera (lower Diptera): evidence of rearrangement following a complete genome duplication in a winter crane fly Genome Biol Evol 2012 4 2 89 101 10.1093/gbe/evr131 22155689 PMC3269971

[B18] Podsiadlowski L Kohlhagen H Koch M The complete mitochondrial genome of *Scutigerella causeyae* (Myriapoda: Symphyla) and the phylogenetic position of Symphyla Mol Phylogenet Evol 2007 45 1 251 260 10.1016/j.ympev.2007.07.017 17764978

[B19] Hall TA BioEdit: a user-friendly biological sequence alignment editor and analysis program for Windows 95/98/NT Nucleic Acids Symp Ser 1999 41 95 98

[B20] Lowe TM Eddy SR tRNAscan-SE: a program for improved detection of transfer RNA genes in genomic sequence Nucleic Acids Res 1997 25 5 955 964 9023104 10.1093/nar/25.5.955 PMC146525

[B21] Stothard P Wishart DS Circular genome visualization and exploration using CGView Bioinformatics 2005 21 4 537 539 10.1093/bioinformatics/bti054 15479716

[B22] Guo X Liu S Liu Y Comparative analysis of the mitochondrial DNA control region in cyprinids with different ploidy level Aquaculture 2003 224 1–4 25 38

[B23] Ravago RG Monje VD Juinio-Menez MA Length and sequence variability in mitochondrial control region of the milkfish, *Chanos chanos* Mar Biotechnol (NY) 2002 4 1 40 50 10.1007/s10126-001-0076-4 14961287

[B24] Mjelle KA Karlsen BO Jorgensen TE Moum T Johansen SD Halibut mitochondrial genomes contain extensive heteroplasmic tandem repeat arrays involved in DNA recombination BMC Genomics 2008 9 10 10.1186/1471-2164-9-10 18186947 PMC2248175

[B25] Manchado M Catanese G Ponce M Funes V Infante C The complete mitochondrial genome of the Senegal sole. *Solea senegalensis* Kaup. Comparative analysis of tandem repeats in the control region among soles DNA Seq 2007 18 3 169 175 17454000 10.1080/10425170701308956

[B26] Shi W Kong XY Wang ZM Jiang JX Utility of tRNA genes from the complete mitochondrial genome of *Psetta maxima* for implying a possible sister-group relationship to the Pleuronectiformes Zool Stud 2011 50 5 665 681

[B27] Zhang XY Yue BS Jiang WX Song ZB The complete mitochondrial genome of rock carp *Procypris rabaudi* (Cypriniformes: Cyprinidae) and phylogenetic implications Mol Biol Rep 2009 36 5 981 991 10.1007/s11033-008-9271-y 18496768

[B28] Ponce M Infante C Jimenez-Cantizano RM Perez L Manchado M Complete mitochondrial genome of the blackspot seabream, *Pagellus bogaraveo* (Perciformes: Sparidae), with high levels of length heteroplasmy in the WANCY region Gene 2008 409 1–2 44 52 18191919 10.1016/j.gene.2007.11.004

[B29] He C Han J Ge L Zhou Z Gao X Mu Y Liu W Cao J Liu Z Sequence and organization of the complete mitochondrial genomes of spotted halibut (*Verasper variegatus*) and barfin flounder (*Verasper moseri*) DNA Seq 2008 19 3 246 255 17852351 10.1080/10425170701563303

[B30] Desjardins P Morais R Sequence and gene organization of the chicken mitochondrial genome. A novel gene order in higher vertebrates J Mol Biol 1990 212 4 599 634 10.1016/0022-2836(90)90225-B 2329578

[B31] Seligmann H Krishnan NM Rao BJ Possible multiple origins of replication in primate mitochondria: alternative role of tRNA sequences J Theor Biol 2006 241 2 321 332 10.1016/j.jtbi.2005.11.035 16430924

[B32] Seligmann H Krishnan NM Mitochondrial replication origin stability and propensity of adjacent tRNA genes to form putative replication origins increase developmental stability in lizards J Exp Zool B Mol Dev Evol 2006 306 5 433 449 16463378 10.1002/jez.b.21095

[B33] Mabuchi K Miya M Satoh TP Westneat MW Nishida M Gene rearrangements and evolution of tRNA pseudogenes in the mitochondrial genome of the parrotfish (Teleostei: Perciformes: Scaridae) J Mol Evol 2004 59 3 287 297 10.1007/s00239-004-2621-z 15553084

[B34] Ki JS Jung SO Hwang DS Lee YM Lee JS Unusual mitochondrial genome structure of the freshwater goby *Odontobutis platycephala*: rearrangement of tRNAs and an additional non-coding region J Fish Biol 2008 73 2 414 428 10.1111/j.1095-8649.2008.01911.x

[B35] Kong X Dong X Zhang Y Shi W Wang Z Yu Z A novel rearrangement in the mitochondrial genome of tongue sole, *Cynoglossus semilaevis*: control region translocation and a tRNA gene inversion Genome 2009 52 12 975 984 10.1139/G09-069 19953125

[B36] Cantatore P Gadaleta MN Roberti M Saccone C Wilson AC Duplication and remoulding of tRNA genes during the evolutionary rearrangement of mitochondrial genomes Nature 1987 329 6142 853 855 10.1038/329853a0 3670390

[B37] Guja KE Garcia-Diaz M Hitting the brakes: termination of mitochondrial transcription Biochim Biophys Acta 2012 1819 9–10 939 947 22137970 10.1016/j.bbagrm.2011.11.004 PMC3408806

[B38] Fernandez-Silva P Enriquez JA Montoya J Replication and transcription of mammalian mitochondrial DNA Exp Physiol 2003 88 1 41 56 10.1113/eph8802514 12525854

[B39] Yakubovskaya E Mejia E Byrnes J Hambardjieva E Garcia-Diaz M Helix unwinding and base flipping enable human MTERF1 to terminate mitochondrial transcription Cell 2010 141 6 982 993 10.1016/j.cell.2010.05.018 20550934 PMC2887341

[B40] Mueller RL Boore JL Molecular mechanisms of extensive mitochondrial gene rearrangement in plethodontid salamanders Mol Biol Evol 2005 22 10 2104 2112 10.1093/molbev/msi204 15987876

[B41] Shah DM Langley CH Complex mitochondrial DNA in *Drosophila* Nucleic Acids Res 1977 4 9 2949 2960 10.1093/nar/4.9.2949 909797 PMC342626

[B42] Raimond R Marcade I Bouchon D Rigaud T Bossy JP Souty-Grosset C Organization of the large mitochondrial genome in the isopod *Armadillidium vulgare* Genetics 1999 151 1 203 210 9872960 10.1093/genetics/151.1.203 PMC1460444

[B43] Clayton DA Smith CA Jordan JM Teplitz M Vinograd J Occurrence of complex mitochondrial DNA in normal tissues Nature 1968 220 5171 976 979 10.1038/220976a0 5701854

[B44] Chang DD Clayton DA Precise assignment of the light-strand promoter of mouse mitochondrial-DNA - a functional promoter consists of multiple upstream domains Mol Cell Biol 1986 6 9 3253 3261 3023972 10.1128/mcb.6.9.3253-3261.1986 PMC367063

