# Supplementary material for: Complete mitogenome sequences of four flatfishes (Pleuronectiformes) reveal a novel gene arrangement of L-strand coding genes
Source: BMC Evol Biol. 2013 Aug 20;13:173. doi: 10.1186/1471-2148-13-173 (PMC3751894; doi:10.1186/1471-2148-13-173)
Supplement: Additional file 1: Table S1 — The primers used for fragment amplification in four flatfish mitogenomes. [file 1471-2148-13-173-S1.docx]

Table S1 The primers used for fragment amplification in four flatfish mitogenomes

|  | Forward  primer | Sequences (5′-3′) |  | Reverse  primer | Sequences (5′-3′) |
| --- | --- | --- | --- | --- | --- |
| *Crossorhombus_azureus* | Z15 | ATTAAAGCATAACHCTGAAGATGTTAAGAT |  | H15149 | AAACTGCAGCCCCTCAGAATGATATTTGTCCTCA |
|  | Z17054 | GYCGGTGGTTARAATCCTCCCTACTGCT |  | F13413 | TAGCTGCTACTCGGATTTGCACCAAGAGT |
|  | QY18-49R | CATGAAACATCGGCGTGG |  | QY18-49F | AGCGCTACGGCAATAAGG |
|  | Z13347 | AAGGATAACAGCTCATCCGTTGGTCTTAGG |  | F-COI | TAAACTTCAGGGTGACCAAAAAATCA |
|  | QY-COI-R | TCTCCCTTCACTTAGCCG |  | QY-COI-F | TCAGTTGCCAAATCCACC |
|  | FISHR1 | TCAACCAACCACAAAGACATTGGCAC |  | F5196 | CTAAATGGTTGGGGTATGG |
|  | Z10818 | TTYGAAGCAGCCGCMTGATACTGACAYTT |  | F2671 | AGATAGAAACTGACCTGGAT |
|  | QY54-96R | TGTAAGAGACCGTGCCTG |  | QYZ15-F | TAACCACTCTTTACGCCG |
|  | 16SAR | CGCCTGTTTATCAAAAACAT |  | F95 | GACAGTAAAGTCAGGACCAAGCCTTTGTGC |
|  |  |  |  | QY54-96F | TCCACTTCAGGGATGTGC |
|  |  |  |  |  |  |
| *Grammatobothus_krempfi* | Z15 | ATTAAAGCATAACHCTGAAGATGTTAAGAT |  | F13413 | TAGCTGCTACTCGGATTTGCACCAAGAGT |
|  | 16SAR | CGCCTGTTTATCAAAAACAT |  | H15149 | AAACTGCAGCCCCTCAGAATGATATTTGTCCTCA |
|  | SXP14-96-R | CATGTGGGTTAGAGTCCC |  | SXP14-96-F | ATGGCCCTGAAGTAGGAG |
|  | FISHR1 | TCAACCAACCACAAAGACATTGGCAC |  | F49 | GGCCCATCTTAACATCTTC |
|  | SXP-COI-R | GGAATCACGATGCTGCTC |  | SXP-Z15-F | TAAGCAAGGCGTTGTGGG |
|  | Z10818 | TTYGAAGCAGCCGCMTGATACTGACAYTT |  | F2671 | AGATAGAAACTGACCTGGAT |
|  | Z13347 | AAGGATAACAGCTCATCCGTTGGTCTTAGG |  | F5196 | CTAAATGGTTGGGGTATGG |
|  | SXP18-49-R | ATCTGCCTCTACCTCCAC |  | SXP-COI-F | GAATGAGTAGGCTGAGGG |
|  | L17114 | RCGCCCAAAGCTAGDATTC |  | FISHF1 | TAGACTTCTGGGTGGCCAAACAATCA |
|  |  |  |  | SXP18-49-F | TAGGACCCTCATCAGTAG |
|  |  |  |  |  |  |
| *Pleuronichthys cornutus* | Z15 | ATTAAAGCATAACHCTGAAGATGTTAAGAT |  | F2753 | TAGATAGAAACTGACCTGGATTACTCCGGT |
|  | Z2625 | GTTTACGACCTCGATGTTGGATCAGGACAT |  | F6746 | GCGGTGGATTGTAGACCCATARACAGAGGT |
|  | 6746L | AGATAGGTAGGCCTCGATCC |  | 6754F | GGTTGGCTTAGTTCTGCYCG |
|  | R-COI-6754 | CTAAGCCATCCTACCTGTG |  | F11089 | TTTAACCAAGACCRGGTGATTGGAAGTC |
|  | Z10818 | TTYGAAGCAGCCGCMTGATACTGACAYTT |  | F13413 | TAGCTGCTACTCGGATTTGCACCAAGAGT |
|  | Z13347 | AAGGATAACAGCTCATCCGTTGGTCTTAGG |  | F17147 | TAGTTTARTGCGAGAATCCTAGCTTTGGG |
|  | L17114 | RCGCCCAAAGCTAGDATTC |  |  |  |
|  |  |  |  |  |  |
| *Platichthys stellatus* | L-Pro | TTCCACCTCTAACTCCCAAAGCTAG |  | H-Phe | CCCATCTTAACATCTTCAGTG |
|  | L-Phe | CTGAAGATGTTAAGATGGG |  | H-16SAR | ATGTTTTTGATAAACAGGCG |
|  | 16SAR | CGCCTGTTTATCAAAAACAT |  | 16SBR | CCGGTCTGAACTCAGATCACGT |
|  | L-123 | ATCAACGAACCAAGTTATCCTA |  | H-123 | TCGGGGAGTCAAGAGAGT |
|  | L-ND2 | ACTTCTAGCTTGAATAGGCCT |  | H-ND2 | AGTTAAGCGGTGGATTGTAG |
|  | L-COI | TCTAGATAGGAAGGCCTTGAT |  | H-COI | AGAAAGTGACAGAGCGGTTAT |
|  | L-CAA | CACCCTTTGAAACACTATCTC |  | H-CAA | AGTGGAATCAGATAGCAAGG |
|  | L-ND34 | CCAAGTGAGATAATGAGCC |  | H-ND34 | CCTAAGACCAACGTATGAGC |
|  | L-ND56 | GTTGAACTGCCTGTAGGTGTA |  | H-ND56 | TGTCCTGGTTTTAGTCCTGG |
|  | L-CytB | GTATATTAAGGCCACTCCTC |  | H-CytB | CTGATGAGTGTTGTGTTCGG |
