# Supplementary material for: Complete mitogenome sequences of four flatfishes (Pleuronectiformes) reveal a novel gene arrangement of L-strand coding genes
Source: BMC Evol Biol. 2013 Aug 20;13:173. doi: 10.1186/1471-2148-13-173 (PMC3751894; doi:10.1186/1471-2148-13-173)
Supplement: Additional file 2: Table S2 — Information of flatfishes used in this study. [file 1471-2148-13-173-S2.docx]

S2 Flatfishes used in this study

| Classification | Species | Accession Nos. | Gene rearrangement |
| --- | --- | --- | --- |
| Pleuronectoidea |  |  |  |
| Bothidae | *Crossorhombus azureus* | This study | YES |
|  | *Grammatobothus krempfi* | This study | NO |
| Pleuronectidae | *Platichthys stellatus* | This study | NO |
|  | *Pleuronichthys cornutus* | This study | NO |
|  | *Kareius bicoloratus* | NC_003176 | NO |
|  | *Verasper variegatus* | NC_007939 | NO |
|  | *Verasper moseri* | NC_008461 | NO |
|  | *Hippoglossus hippoglossus* | NC_009709 | NO |
|  | *Hippoglossus stenolepis* | NC_009710 | NO |
|  | *Reinhardtius hippoglossoides* | NC_009711 | NO |
| Paralichthyidae | *Paralichthys olivaceus* | NC_002386 | NO |
| Scophthalmidae | *Scophthalmus maximus* | NC_013183 | NO |
