# Supplementary material for: Complete mitogenome sequences of four flatfishes (Pleuronectiformes) reveal a novel gene arrangement of L-strand coding genes
Source: BMC Evol Biol. 2013 Aug 20;13:173. doi: 10.1186/1471-2148-13-173 (PMC3751894; doi:10.1186/1471-2148-13-173)
Supplement: Additional file 3: Table S3 — Organization of four flatfishes mitochondrial genomes. [file 1471-2148-13-173-S3.docx]

Table S 3a: Organization of *C. azureus* mitochondrial genome

| Gene | Start/stop  anticodon | Start | End | Orient | Intergenic region |
| --- | --- | --- | --- | --- | --- |
| *tRNA-Phe (F)* | UUC | 1 | 68 | H | 0 |
| *12S* |  | 69 | 1034 | H | 0 |
| *tRNA-Val (V)* | GUA | 1035 | 1105 | H | 0 |
| *16S* |  | 1106 | 2818 | H | 0 |
| *tRNA-Leu (L_1_)* | UUA | 2819 | 2891 | H | 0 |
| *ND1* | ATG/TAA | 2892 | 3869 | H | 0 |
| *tRNA-Ile (I)* | AUC | 3870 | 3938 | H | 0 |
| *tRNA-Met (M)* | AUG | 3946 | 4016 | H | 7 |
| *ND2* | ATG/TAA | 4017 | 5066 | H | 0 |
| *tRNA-Trp (W)* | UGA | 5065 | 5134 | H | -2 |
| *tRNA-Asn (N)* | AAC | 5143 | 5215 | L | 8 |
| *COI* | ATA/TAA | 5223 | 6779 | H | 7 |
| *COII* | ATA/T | 6804 | 7545 | H | 24 |
| *tRNA-Lys (K)* | AAA | 7547 | 7618 | H | 1 |
| *ATP8* | ATG/TAA | 7620 | 7787 | H | 1 |
| *ATP6* | ATG/TAA | 7778 | 8461 | H | -10 |
| *COIII* | ATG/TAA | 8461 | 9246 | H | -1 |
| *tRNA-Gly (G)* | GGA | 9246 | 9316 | H | -1 |
| *ND3* | ATG/T | 9317 | 9665 | H | 0 |
| *tRNA-Arg (R)* | CGA | 9666 | 9734 | H | 0 |
| *ND4L* | ATG/TAA | 9735 | 10031 | H | 0 |
| *ND4* | ATG/T | 10025 | 11405 | H | -7 |
| *tRNA-His (H)* | CAC | 11406 | 11474 | H | 0 |
| *tRNA-Ser (S_2_)* | AGC | 11474 | 11547 | H | -1 |
| *tRNA-Leu (L_2_)* | CUA | 11548 | 11620 | H | 0 |
| *ND5* | ATG/TAG | 11621 | 13459 | H | 0 |
| *Cytb* | ATG/T | 13472 | 14612 | H | 12 |
| *tRNA-Thr (T)* | ACA | 14613 | 14685 | H | 0 |
| *tRNA-Asp (D)* | GAC | 14726 | 14793 | H | 40 |
| CR |  | 14794 | 15480 | H | 0 |
| *tRNA-Gln (Q)* | CAA | 15481 | 15551 | L | 0 |
| *tRNA-Ala (A)* | GCA | 15557 | 15626 | L | 5 |
| *tRNA-Cys (C)* | UGC | 15632 | 15697 | L | 5 |
| *tRNA-Tyr (Y)* | UAC | 15699 | 15769 | L | 1 |
| *tRNA-Ser (S_1_)* | UCA | 15771 | 15841 | L | 1 |
| *ND6* | ATG/TAG | 15868 | 16389 | L | 26 |
| *tRNAL-Glu (E)* | GAA | 16390 | 16457 | L | 0 |
| *tRNAL-Pro (P)* | CCA | 16460 | 16527 | L | 2 |

Table S 3b: Organization of *G. krempfi* mitochondrial genome

| Gene | Start/stop  Anticodon | Start | End | Orient | Intergenic region |
| --- | --- | --- | --- | --- | --- |
| *tRNA-Phe (F)* | UUC | 1 | 69 | H | 0 |
| *12S* |  | 70 | 1025 | H | 0 |
| *tRNA-Val (V)* | GUA | 1026 | 1096 | H | 0 |
| *16S* |  | 1097 | 2827 | H | 0 |
| *tRNA-Leu (L_1_)* | UUA | 2828 | 2900 | H | 0 |
| *ND1* | ATG/TAG | 2917 | 3870 | H | 16 |
| *tRNA-Ile (I)* | AUC | 3877 | 3947 | H | 6 |
| *tRNA-Gln (Q)* | CAA | 3948 | 4018 | - | 0 |
| *tRNA-Met (M)* | AUG | 4018 | 4086 | H | -1 |
| *ND2* | ATG/TAA | 4087 | 5133 | H | 0 |
| *tRNA-Trp (W)* | UGA | 5133 | 5204 | H | -1 |
| *tRNA-Ala (A)* | GCA | 5206 | 5274 | L | 1 |
| *tRNA-Asn (N)* | AAC | 5276 | 5348 | L | 1 |
| *tRNA-Cys (C)* | UGC | 5386 | 5450 | L | 37 |
| *tRNA-Tyr (Y)* | UAC | 5451 | 5517 | L | 0 |
| *COI* | GTG/T | 5519 | 7064 | H | 1 |
| *tRNA-Ser (S_1_)* | UCA | 7066 | 7136 | L | 1 |
| *tRNA-Asp (D)* | GAC | 7145 | 7215 | H | 8 |
| *COII* | ATG/T | 7223 | 7913 | H | 7 |
| *tRNA-Lys (K)* | AAA | 7914 | 7986 | H | 0 |
| *ATP8* | ATG/TAA | 7988 | 8155 | H | 1 |
| *ATP6* | ATG/TAA | 8146 | 8829 | H | -10 |
| *COIII* | ATG/TAA | 8829 | 9614 | H | -1 |
| *tRNA-Gly (G)* | GGA | 9614 | 9685 | H | -1 |
| *ND3* | ATG/T | 9686 | 10034 | H | 0 |
| *tRNA-Arg (R)* | CGA | 10035 | 10103 | H | 0 |
| *ND4L* | ATG/TAA | 10104 | 10400 | H | 0 |
| *ND4* | ATG/T | 10394 | 11774 | H | -7 |
| *tRNA-His (H)* | CAC | 11775 | 11843 | H | 0 |
| *tRNA-Ser (S_2_)* | AGC | 11844 | 11910 | H | 0 |
| *tRNA-Leu (L_2_)* | CUA | 11914 | 11986 | H | 3 |
| *ND5* | ATG/TAA | 11989 | 13827 | H | 2 |
| *ND6* | ATG/AGG | 13823 | 14344 | L | -5 |
| *tRNA-Glu (E)* | GAA | 14345 | 14412 | L | 0 |
| *Cytb* | ATG/TAA | 14424 | 15563 | H | 11 |
| *tRNA-Thr (T)* | ACA | 15565 | 15636 | H | 1 |
| *tRNA-Pro (P)* | CCA | 15637 | 15708 | L | 0 |
| CR |  | 15709 | 16599 | H | 0 |

Table S 3c: Organization of *P. stellatus* mitochondrial genome

| Gene | Start/stop  Anticodon | Start | End | Orient | Intergenic region |
| --- | --- | --- | --- | --- | --- |
| *tRNA-Phe (F)* | UUC | 1 | 68 | H | 0 |
| *12S* |  | 69 | 1017 | H | 0 |
| *tRNA-Val (V)* | GUA | 1018 | 1090 | H | 0 |
| *16S* |  | 1091 | 2804 | H | 0 |
| *tRNA-Leu (L_1_)* | UUA | 2805 | 2878 | H | 0 |
| *ND1* | ATG/TAG | 2879 | 3853 | H | 0 |
| *tRNA-Ile (I)* | AUC | 3859 | 3929 | H | 5 |
| *tRNA-Gln (Q)* | CAA | 3929 | 3999 | L | -1 |
| *tRNA-Met (M)* | AUG | 3999 | 4067 | H | -1 |
| *ND2* | ATG/T | 4068 | 5112 | H | 0 |
| *tRNA-Trp (W)* | UGA | 5113 | 5184 | H | 0 |
| *tRNA-Ala (A)* | GCA | 5186 | 5254 | L | 1 |
| *tRNA-Asn (N)* | AAC | 5256 | 5328 | L | 1 |
| *tRNA-Cys (C)* | UGC | 5367 | 5431 | L | 38 |
| *tRNA-Tyr (Y)* | UAC | 5432 | 5499 | L | 0 |
| *COI* | GTG/TAA | 5501 | 7060 | H | 1 |
| *tRNA-Ser (S_1_)* | UCA | 7061 | 7131 | L | 0 |
| *tRNA-Asp (D)* | GAC | 7146 | 7216 | H | 14 |
| *COII* | ATG/T | 7223 | 7913 | H | 6 |
| *tRNA-Lys (K)* | AAA | 7914 | 7986 | H | 0 |
| *ATP8* | ATG/TAA | 7988 | 8155 | H | 1 |
| *ATP6* | ATG/TAA | 8146 | 8829 | H | -10 |
| *COIII* | ATG/TAA | 8829 | 9614 | H | -1 |
| *tRNA-Gly (G)* | GGA | 9615 | 9686 | H | 0 |
| *ND3* | ATG/T | 9686 | 10034 | H | -1 |
| *tRNA-Arg (R)* | CGA | 10036 | 10104 | H | 1 |
| *ND4L* | ATG/TAA | 10104 | 10400 | H | -1 |
| *ND4* | ATG/T | 10394 | 11774 | H | -7 |
| *tRNA-His (H)* | CAC | 11775 | 11844 | H | 0 |
| *tRNA-Ser (S_2_)* | AGC | 11845 | 11911 | H | 0 |
| *tRNA-Leu (L_2_)* | CUA | 11916 | 11988 | H | 4 |
| *ND5* | ATG/TAA | 11989 | 13827 | H | 0 |
| *ND6* | ATG/TAG | 13824 | 14345 | L | -4 |
| *tRNA-Glu (E)* | GAA | 14346 | 14414 | L | 0 |
| *Cytb* | ATG/T | 14419 | 15559 | H | 4 |
| *tRNA-Thr (T)* | ACA | 15560 | 15632 | H | 0 |
| *tRNA-Pro (P)* | CCA | 15633 | 15703 | L | 0 |
| CR |  | 15704 | 17103 | H | 0 |

Table S 3d: Organization of *P. cornutus* mitochondrial genome

| Gene | Start/stop  Anticodon | Start | End | Orient | Intergenic region |
| --- | --- | --- | --- | --- | --- |
| *tRNA-Phe (F)* | UUC | 1 | 68 | H | 0 |
| *12S* |  | 69 | 1014 | H | 0 |
| *tRNA-Val (V)* | GUA | 1015 | 1088 | H | 0 |
| *16S* |  | 1089 | 2798 | H | 0 |
| *tRNA-Leu (L_1_)* | UUA | 2799 | 2872 | H | 0 |
| *ND1* | ATG/TAG | 2873 | 3847 | H | 0 |
| *tRNA-Ile (I)* | AUC | 3854 | 3924 | H | 6 |
| *tRNA-Gln (Q)* | CAA | 3924 | 3994 | L | -1 |
| *tRNA-Met (M)* | AUG | 3994 | 4062 | H | -1 |
| *ND2* | ATG/TAA | 4063 | 5109 | H | 0 |
| *tRNA-Trp (W)* | UGA | 5109 | 5180 | H | -1 |
| *tRNA-Ala (A)* | GCA | 5183 | 5251 | L | 2 |
| *tRNA-Asn (N)* | AAC | 5253 | 5325 | L | 1 |
| *tRNA-Cys (C)* | UGC | 5364 | 5427 | L | 38 |
| *tRNA-Tyr (Y)* | UAC | 5428 | 5495 | L | 0 |
| *COI* | ATG/TAA | 5497 | 7047 | H | 1 |
| *tRNA-Ser (S_1_)* | UCA | 7048 | 7118 | L | 0 |
| *tRNA-Asp (D)* | GAC | 7134 | 7204 | H | 15 |
| *COII* | ATG/T | 7213 | 7903 | H | 8 |
| *tRNA-Lys (K)* | AAA | 7904 | 7976 | H | 0 |
| *ATP8* | ATG/TAA | 7979 | 8146 | H | 2 |
| *ATP6* | ATG/TAA | 8137 | 8820 | H | -10 |
| *COIII* | ATG/TAA | 8820 | 9605 | H | -1 |
| *tRNA-Gly (G)* | GGA | 9605 | 9676 | H | -1 |
| *ND3* | ATG/TAA | 9677 | 10027 | H | 0 |
| *tRNA-Arg (R)* | CGA | 10026 | 10094 | H | -2 |
| *ND4L* | ATG/TAA | 10095 | 10391 | H | 0 |
| *ND4* | ATG/T | 10385 | 11765 | H | -7 |
| *tRNA-His (H)* | CAC | 11766 | 11835 | H | 0 |
| *tRNA-Ser (S_2_)* | AGC | 11838 | 11901 | H | 2 |
| *tRNA-Leu (L_2_)* | CUA | 11906 | 11978 | H | 4 |
| *ND5* | ATG/TAA | 11979 | 13817 | H | 0 |
| *ND6* | ATG/TAG | 13814 | 14335 | L | -4 |
| *tRNA-Glu (E)* | GAA | 14336 | 14403 | L | 0 |
| *CytB* | ATG/T | 14408 | 15548 | H | 4 |
| *tRNA-Thr (T)* | ACA | 15549 | 15621 | H | 0 |
| *tRNA-Pro (P)* | CCA | 15621 | 15691 | L | -1 |
| CR |  | 15692 | 17469 | H | 0 |
