# Supplementary material for: Complete mitogenome sequences of four flatfishes (Pleuronectiformes) reveal a novel gene arrangement of L-strand coding genes
Source: BMC Evol Biol. 2013 Aug 20;13:173. doi: 10.1186/1471-2148-13-173 (PMC3751894; doi:10.1186/1471-2148-13-173)
Supplement: Additional file 4: Figure S4 — Gene maps of the mitochondrial genome of G. krempfi, P. cornutus and P. stellatus. [file 1471-2148-13-173-S4.docx]

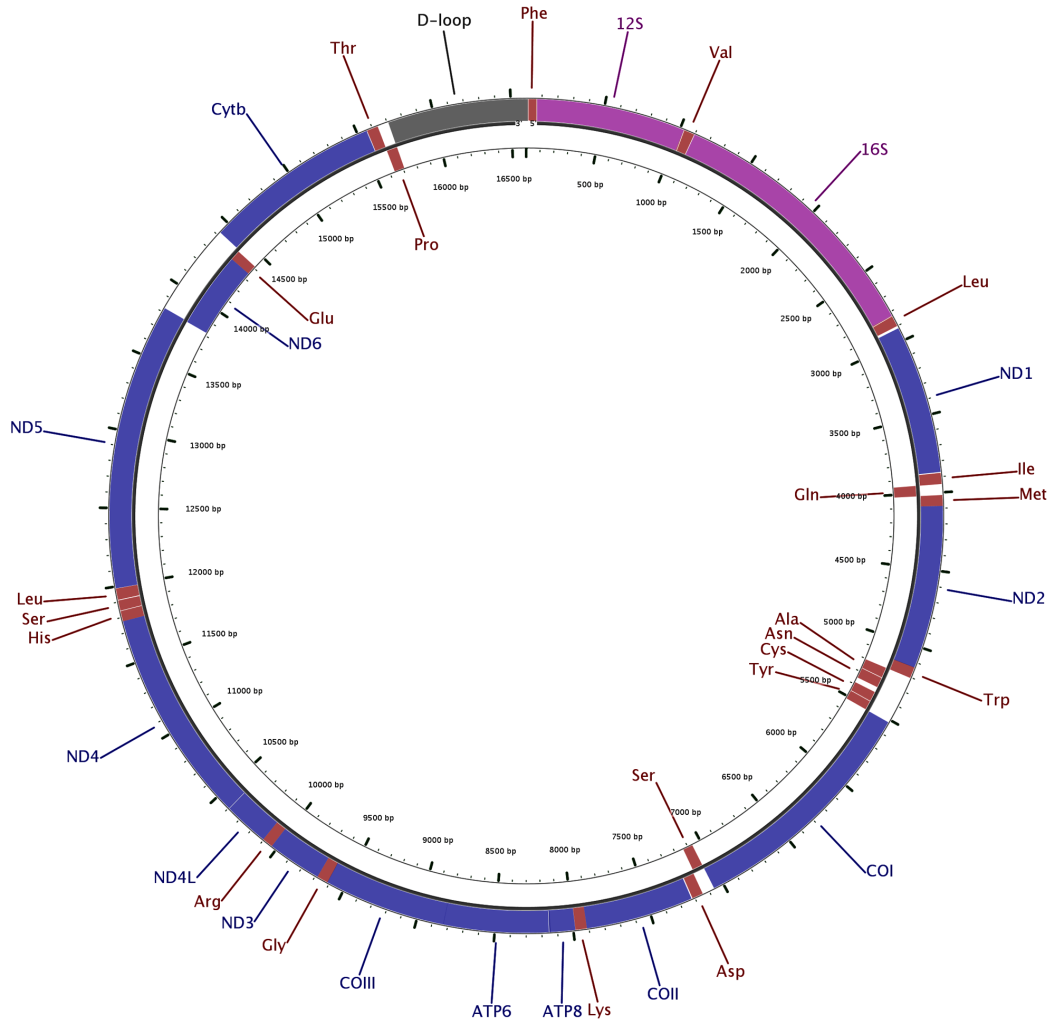


Figure S4 a Gene map of the mitochondrial genome of *G. krempfi*.


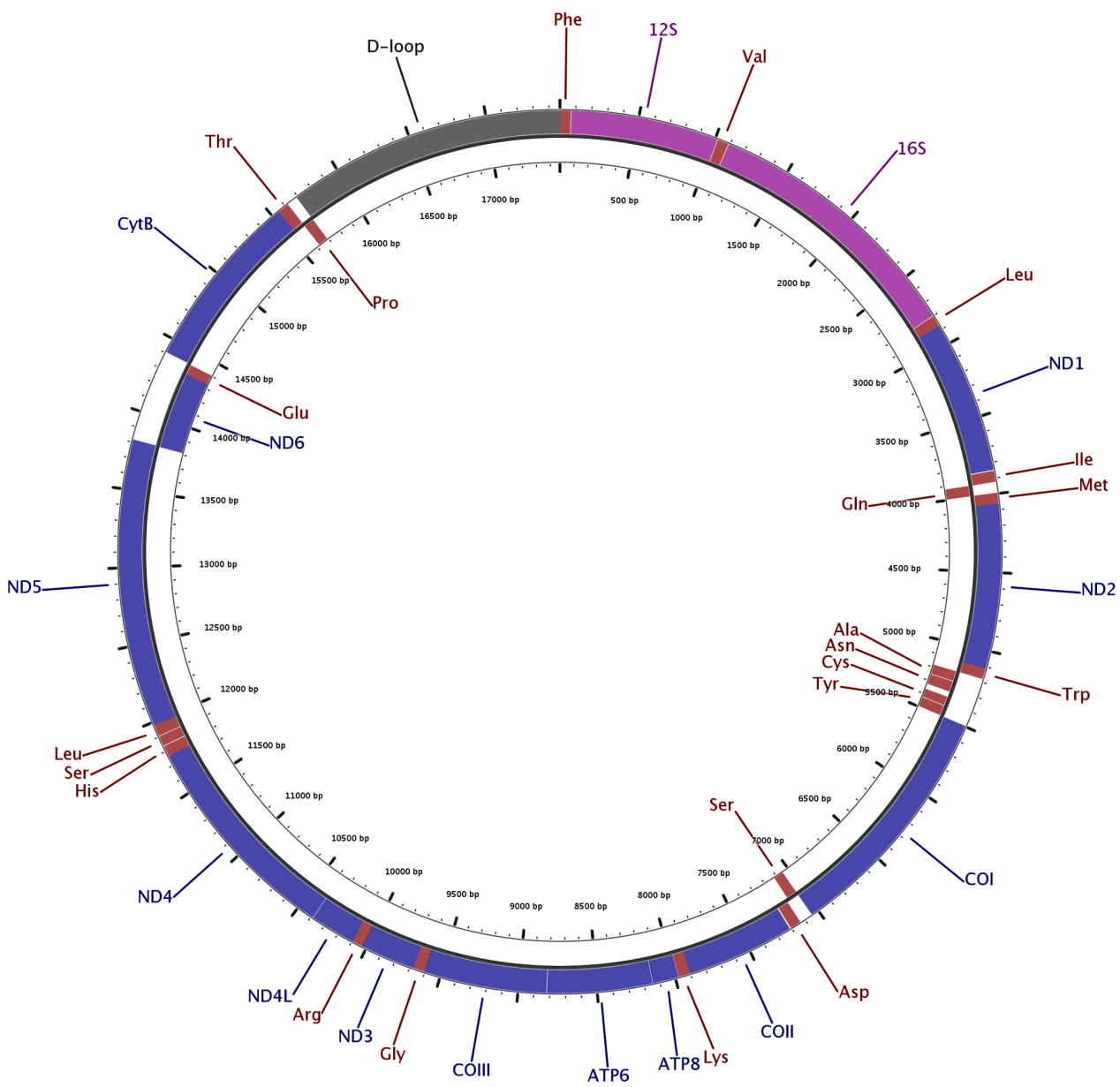


Figure S4 b Gene map of the mitochondrial genome of *P. cornutus*.


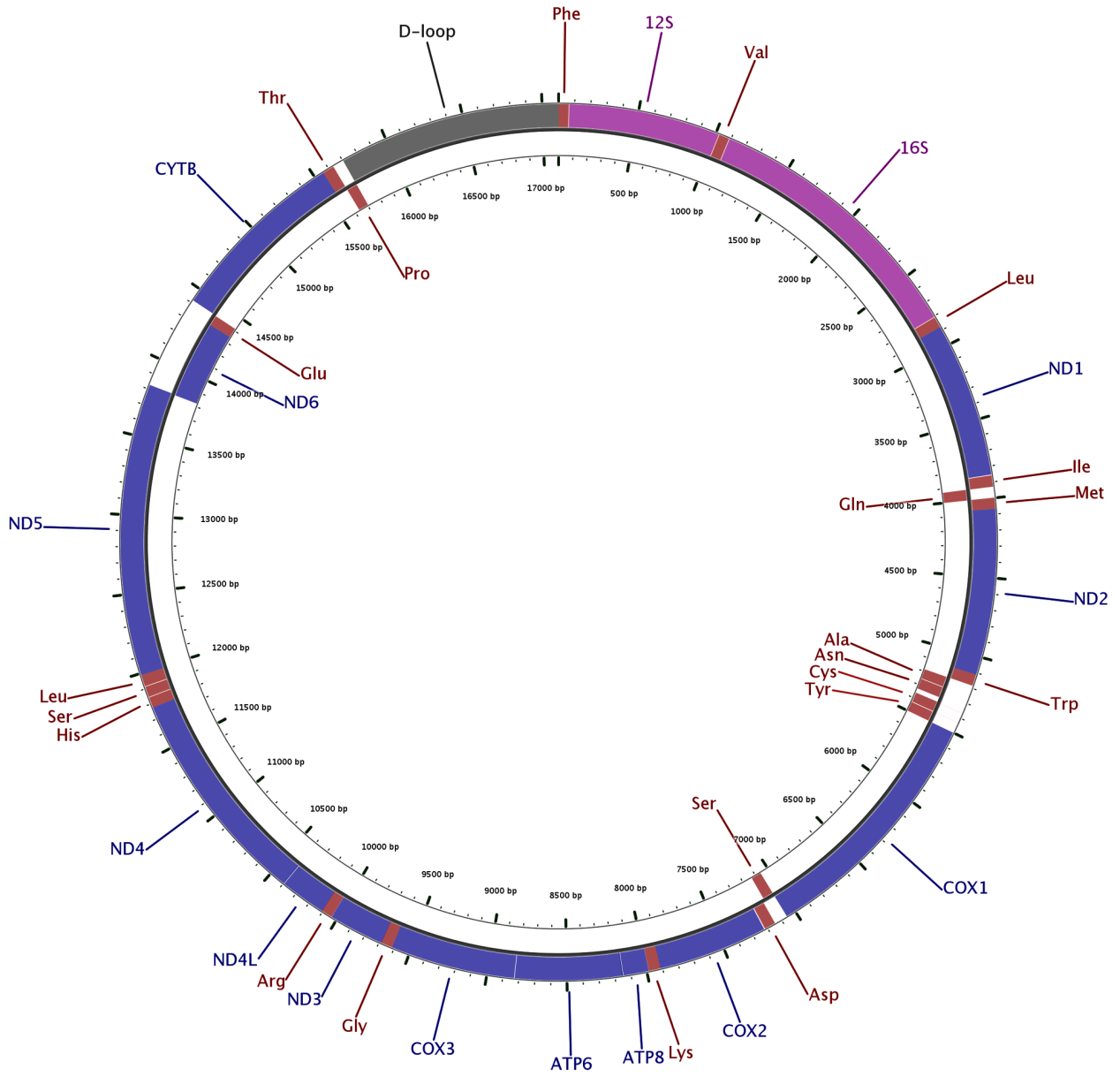


Figure S4 c Gene map of the mitochondrial genome of *P. stellatus.*
