# Supplementary material for: Complete mitogenome sequences of four flatfishes (Pleuronectiformes) reveal a novel gene arrangement of L-strand coding genes
Source: BMC Evol Biol. 2013 Aug 20;13:173. doi: 10.1186/1471-2148-13-173 (PMC3751894; doi:10.1186/1471-2148-13-173)
Supplement: Additional file 5: Figure S5 — Complete CR DNA fragments alignment of 11 Pleauonectoidea fishes. [file 1471-2148-13-173-S5.docx]

**TAS1 TAS-cTAS box TAS2**

V.var TTTATGTGCA T—ATATGTA ATAACACCAT ATATTTATAG TAACCATTTT ATGTGA-TGT ACTAAGACAT TCATGTATT- ATAACCTAAT CTAGTAATAT AGCACTCATT CACCAACATT TTTAACTAAA GTAA-ACTAA [140]

V.mos .......A.. .--....... .......... .......... .........C ......-... .TC.G..... ........A- .......... ......T... .......... ..T....... ......C..G ....-..... [140]

H.hip ..C..A.A.. .--G...... .......... .......... .......... ......-... ....G..... A.......A- ..C...A... ..C......C .......... ..T....... .........G A..G-..... [140]

H.ste ..C..A.A.. .--G...... .......... .......... .......... ....A.-... ....G..... A.......A- ..C...AC.. ..C......G .A........ .....C.... .........G ACG.-..... [140]

R.hip .....A.A.. .--G...... T......... .......... .......... G...A.-... ....G..... A.......A- ..C......C A......... .......... ..T..C.... .........G AA.TT....G [140]

P.ste ..C....A.. .--G...... .......... --------.. .......... ..A.A.-..A ....G..... ........A- .......... .......... .......... ..A....... .........G A..G-....G [140]

P.oli ..A..A.A.. .--....... ..T....... .......... ...A....AA G.CC..-... ..A---.G.C A..AA.GGAT G.G.A.A..A .AT.GTG.CA .A..T....A T....G.TA. A-......-. A..TATAC.. [140]

S.max ....-A.A.. .--....... T......... TA..C....T .........A GACC.G-... C.GTCA.... .AG..AT.A- ..C.GTA..G TAT.G.G.TA .A..T...AG -.T....TAC CGG.CT.CT. .GGC-..ATG [140]

P.cor .....AGA.. .--....... ..T....... .......... .......... ..ACAG-... .TCGG..... .T.....A.- ..C.G...TA AC...TT.TA .A..AA...G -CT.GGT.A. .CAC.....G ...G-..A.. [140]

G.kre .A...A.... .CC.CG...G GGT...TAT. ..G.A.-.G. ..CAT.A.A. G.A.A.A.T. ..AT.C.T.C .A.A....GT ..GTA.A..A TACA..C.TG GTTTT.TGCA TGG.GTTTA. AA.GGA..C. AC.GACT... [140]

C.azu ---------- ---...CCAG GCTCTG...C TC.AAC.C.A ..-T..CGAC G.CG..ACA. ..A.T.TA.. .TCACC...- -C..TTAT.. .A..C.T.CA ..TTTAG.CA .GTT.--TG. AA.G.TCGG. AA.G-G.AC. [140]

V.var AACC-TAATT AATCACTAAT TT---TAAAT ATGTAAAACT CTAGGACCA- AT-CGAAATT TAAGACCGAA CACGACACT- CATCAGTTAA GTTATACCAA GACTCAAAAT CTCGCCCAT- --CATAAATT CCTATGTAGT [280]

V.mos ....-...C. .....T.G.. ..---..... ..A.G...T. .C.....T.- G.-.....C. .......... .........- .......C.. .......... .......... ......A.CA --..C..... .......... [280]

H.hip ....-.G.A. .......... C.---..... ....G...G. .C.......- G.-......C .......... ...A.....- .......CG. .......... .......... ......G.C- --.CC...A. .......... [280]

H.ste ....-.G.G. G......... C.---..... .A..G...G. .C.......- G.-...G..C .......... ...A.....- .......CG. .......... .......... ......G.C- --.TC...A. T......... [280]

R.hip ....C.G.C. TCA..T.C.C G.---..C.. ....G...T. .C.......- GC-...C... .......... ...A.....- ..C.G..C.. .......... .......... T..A.T..C- --TTA...AA ..A.CCC.A. [280]

P.ste ....-C...C CCC....G.C C.---..C.. TAC.GG.... A.G..G...- GC-...G... .......... ...A.....- .......CG. .......... .......... ...-TT..C- --ATC.G..C .......... [280]

P.oli ..A.CA---A .CCT.TA.GG .A---..C.A TAA-.G..T- TA.A...T.- G.-.....C. .T.C...... ...A..CT.- ...AT..C.. .......... ........C. TCT.T.G..C ---CC..... ..C...C... [280]

S.max ..T.--..AA .TA..A.... CA---CC.TG ..AC.G..-. .G...G.A.- G.-.....AC C....T.A.. TCAATT.T.- ....C..A.. ........TT T.....CC.. .CTATTG.C- --.TC..TCC ..G....... [280]

P.cor ....--..CG .T-TGAA... CG---..... TAAC.G..-A T..A.....- ..-...G... .......T.. .TAATT.T.- ..C....... .A........ .......... .C..A.AT.- --TTG...AA TA........ [280]

G.kre ..TTTAC..A ..A..T...C .AATTC.C.G CCA..GT.TG TA.A.GTA.- ..G...T... .....T.TC. GTTA.A.TAG ...ATACC.T .A..C....G .......... TATATGT.CA TGACC..... T--.CCC.A. [280]

C.azu TC..TG..G. GGA...AC.C A.---AGG.G TA.G..C..A .GTA.G.A.A GCC.A.C..A .T.A..AAG. GGGA..C.AC ..CGT-...G ..CGC.T.C. TG.----..C ...------- ----C..TG. TG.GATC... [280]

V.var AAGAGCCTAC CAACCGGTG- ATTTCTGAAT GATAACTCTT ATTGATGGTC AGGGACAGAA ATCGTGGGGG TTTCACTCAG TGAACTATTC CTGGCATTTG GTTCCTACTT CAGGGCCAT- TTATCGATAT TATCCCTCAC [420]

V.mos .......... .........- ......T... .......... .......... .......... .......... .......... .......... .......... .......... .........- .G.CT..... .......... [420]

H.hip .......... .........- ...C..T... .......... .....G...G .......A.. ..T....... ........G. ....T..... .......... .......... .........- .G..T...G. .......... [420]

H.ste .......... .........- ...C..T... .......... .....G...G .......A.. ..T....... ........G. ....T..... .......... .......... .........- .G..T..... .......... [420]

R.hip .......... .........- ..CC..T... .......... .....G...G .......A.. ..T....... ........G. ....T..... .......... .......... .........- .G..T..... .......... [420]

P.ste .......... .........- ...A..T... ......GG.. .....A...G .......A.. ..T....... ......CTC. ...TT..... .......... .......... .........- GA.....-G. C.CT..C... [420]

P.oli .......... ..T.A.T..- ...A..T... .CC...GG.. .....A...G .......A.. ..T....... ......AT.. .......... .......... .......... .........- GACCT..... ...T...... [420]

S.max ....A..... ...A.T...- ......T... ......GG.. .....A...G .......AG. .C........ CGG...CTCT ..C....... .......... .C........ ....T....- .ACCT..... ...T..C.C. [420]

P.cor .......... .........- ...C..T... ......GG.. .....A...G .......A.. ..T....... ........G. ....T..... .......... .......... ....TT.TA- .A.CT.G... C......... [420]

G.kre .....AA.CG ..T.G.T..- ..AA..T.CC ......GG.. .......... .A........ CGT....... .......TCT .......... .......A-A .C........ .........- .G..T.C--C ....G...C. [420]

C.azu ....A..G.G .T..AA..AC ...C..T... ......GG.. .......... .A.....AG. CCT....... .....A.TCT A.C.T..... .......... .......... ....AA...C .C.C.....C A.....-..T [420]

V.var ACTTTCATCG ACGCTTGCAT AAGTTAATGT TGATAATACA TACGACTCGT TACCCAGCAA GCCGGGCGTT CACTCCAGCG GGTAAGGGGT TC-TCTTTTT TTTT-TTCCT TTCATCTGGC ATTACAGAGT GCATCCAGCC [560]

V.mos .......... ..A....... .......... .......... .......... .......... .......... .......... .......... ..-....... ....-..... .......... .......... .......... [560]

H.hip .......... ..A...A... .......... .......... .......... ......C... .......... .......... .......... ..-....... ....-..... ....CT..A. ...T..A... .......... [560]

H.ste .......... ..A...A... .......... .......... .......... .......... .......... .......... .......... ..-....... ....-..... ....CT..A. T..T..A... .......... [560]

R.hip .......... ..A...A... .......... .......... .......... ......C... .......... .......... .......... ..-....... ....-..... ....CT..A. ...T...... .......... [560]

P.ste .......... ......A... .......... .......... .......... .......... .......... .......... .......... ..-....... ....-.-... ....CT.... ...T...... .......... [560]

P.oli .......... ..A...A... .......... ..G..T.... .......... .......... .......... .......... .......... ..-....... ....-.C.T. .......... ...T...... .....T.... [560]

S.max .......... ......A... .........G ..GA..C-.. ..-ACT.... .....CC... ....A..... .T...T..G. ...C.A.A-. ..-.T..... ....C..... ...GCTG.T. ...T..C... ...G.AG.G. [560]

P.cor .......... ..A..GA... .......... ..G....... .......... ......C... .......... .......... .......... ..-....... ....T..... ....CTC... ...T...... .......... [560]

G.kre .....T.... ......A... TGAC.G.... ...GGT.... .G-AC..... ......C... .......... ........T. A.C....... ..-....A.. ....-...T. ...C...T.. ...T...... .......... [560]

C.azu ..G.C...T. ..A....... .........G ...C..C-.. .-.TCT.... .....CC... .......A.. ..T..T.AA. TAC.TCT... ..C....... C.C.T..... ....C.CT.. ..----.G.C CTGGTTGTT. [560]

V.var AAGAGAAACG TTTAAAGGGT GAGCATTTTT CTTGCTCGCG GCGTACATAG TATCCATG-T AACAAGTCTT TATTAGAAGA ATAACATTAA AGGATATCAT GTGCATAAAA ATATGCTCAT TTATCTTGTC TTCCCCAGGA [700]

V.mos ..A....... .......... .......... .......... .......... ...T....-. ..T....... .......... .......... G......... .........G .......... ....TC..C. .......... [700]

H.hip ..CG..G... .......... .....C.... .....A.... C......... .....G..-. ..TG...... .........G .......... GT.T...... .........G ..T....... ..C.TC.... A......... [700]

H.ste ..C...G... .........G .....C.... .....A.... C......... ........-. .GGG...... .....A..A. .......... GT.T...... .........G ..T....... ..C.TC.A.. A......... [700]

R.hip ..C...G... .......... .....C.... .....A.T.A .......... ........-. T.T....... .........G .......A.. G......... ........G. ..G.....C. ..C.TC.A.. A......... [700]

P.ste ..CC...... .......... .......... .....A.T.. C......... ........-. ...G.AA... .......... .CC....... TA.......A ........GG ..G....TG. ....TC.AC. .......... [700]

P.oli G.T.....TA ..C-....TG ..A.....CC T.GCG.GCAA --...A.... .C..A...TA .TT..T-... ...CTA.... .......... .A.......A ........GG G.GGT..TG. .A...CAAAG A....TTA.. [700]

S.max --TT.--.TA .CCT....TG ..A....CC. ..GAA.TCA. --.A.A.... ...TA...A. CTAG..G... .CGACA.... .CC....A.C -T...T...C .A......G- G.GCTACTGC ..C..C.ACG ACT..T.A.. [700]

P.cor ..CCA--..C G..C....T. .TA......C .....C..G. --.G.A.... ........A. .-AT..A... .........G ...C...A.. GT..A....A ........GT ..G.C..TG. ..C..C.A.. .....T..AG [700]

G.kre ..T....... ..C-....TC .GA.---.C. ACGAA.-TA. --T..T.A.A ATGTA...AA TTA...A.A. G.ACT....T C.T...A-.. CT...T...A .......C.T T..CTTGT.. .C.A.AAACA A.ATTTTAA. [700]

C.azu ..TTA.CC.A C---....TC .GA...AC.. TGATTGTC.C TGC...TA.T ..GTG.ATG. T.A...A.AA .C.ACA.GAG CAG....AT. .AT..T...A .GA.....G- --GCTA..G. ....T....T CGTAA.TA.G [700]

**CSB-2 CSB-3**

V.var TACCCCCTTT TTC--GCGCG TAAAACCCCC CCTACCCCCC TAAACTCCTG AAGTTGCTAA CACTCCTGAA AACCCCCCGG AAACAGGACA AACCTCTAAT AGCTCAGAAA AGGCAATAAC TTTTCAAC-- ---------- [840]

V.mos .......... ..T--..... .......... .......... C......... .......... .......... .......... .......... ........G. ........G. .A.....T.. ........GT ACTTTTCAAC [840]

H.hip .......... ..T--..... C......... .-........ .....C.... .......... G..C...... .......... .......... .......GG. .......... G......T.. ........C- ACTTTTCAAC [840]

H.ste .......... ..TT-..... A......... .-........ .....C.... .......... G..C...... .......... .......... .......GG. .......... .......T.. ....AT..C- ACTTTTCAAC [840]

R.hip .......... ..T---.... .......... .-........ .......... .......... G..C...... .......... .......... .......GG. ....T..... .......T.. ........CT ACTTTTCAAC [840]

P.ste .G........ ..TGC..... A..G...... .-........ ..C..C.... .......... G..C...... .......-.. .......... ...T...GG. ....T...-. ..AT..CT.. ..--G...-- ---------- [840]

P.oli .CA....C.. ..TG--.... C......... .-CCA..... C........A .G...ATCT. T......... .......... ........A. .C..CGA.GC .T....CTCT TCATCTC.C. C.AA..TGGG TTAGACTTAC [840]

S.max .TA....C.- ..TTT..... G......... .......... CT.......A .G........ .......... .......... ........-. .G..C...G. ..T.TTT.-- TTCTG...C. A.A.ACGTA- ---TCCTCGC [840]

P.cor .TG....GGG ..TTC..... -....T.... .......... A......G.. ......T..T G.TC.TGA.. .C........ ........-. ...T....G. .ATGGGT.-- CCC.---CC. C..CGGG.C- ---TTTTGGC [840]

G.kre .-------.. ...------- -----..... .-.T...... C...A.AT.A .---.AAGGG A.TC.T.--. ....G...CC ..T.C..T.T .TT----..C .T...CCTT. TTAACT.TCT CCAA---GGG AAACCCCTAC [840]

C.azu C.------C. ..T------- ----G..... ...C.....A A..CAA.A.A .TA.C----- ---------- ---------- ---------- ---------- ---------- ---------- ---------- ---------- [840]

V.var CCTAAAATAC CACTAGTAAA CCACAAGCCC ACCAGTTGTT TTAATAATGG A--------- ---------- ---------- ---------- ---------- ---------- ---------- ---------- ---------- [980]

V.mos .........T .....A.C.. ...T....T. .......... .......... T--------- ---------- ---------- ---------- ---------- ---------- ---------- ---------- ---------- [980]

H.hip .--------- ---------- ---------- ---------- ---------- ---------- ---------- ---------- ---------- ---------- ---------- ---------- ---------- ---------- [980]

H.ste ..AC...... .C..G.CTT. T.....C... .......... ........AC CACTTTTCAA CCCACAAATA CCCCTGGCTT ATCACAACCC CACCAGTTGT TTTAATAATA CCACTTTTCA ACCCACAAAT ACCCCTGGCT [980]

R.hip .......... .C..GACC.. AG.T...... .......A.. .......... TACTTTTCAA CCCTAAAATA CCCCTGACCA AAGATAAGCC CACCAGTTAT TTTAATAATG GTACTTTTCA ACCCTAAGAT ACCCCTGACC [980]

P.ste .........A ..GCG..CCG ..C..GA.TT T...-..A.. AC..C.-.AT T--------- ---------- ---------- ---------- ---------- ---------- ---------- ---------- ---------- [980]

P.oli ...C..CCTT TC.CCCATTT TA.TG.TT.A ..TTC.CTCA C.CT.C..CT CACCCTAACA TGGGTTAGAC TTACCCTCAA CCTTTCCCCC ATTTTAATGA TTCAACTTCT CTCACTCTTC ATCTCACCCT AACATGGGTT [980]

S.max .....C..-- ----....T. GAGTTTA.TG TTT.TA.ACA G..T..TATA CAGTATTATA TA--CAGTAT TATATACAGT ATTATATACA GTATTATATA C---AGTATT ATAT----AC AGTATTATAT A--CAGTATT [980]

P.cor .....T..T- ---..TA.T. .A....ATTG GTTT...... GC.T..TATA GTGTTTTGTA AAAGCACAAA CAAGCACATC TTTATTCCCC AAGCCAAATG GCCGGGAACT AGATCTTTAA GCTATTATAA ATCCAATATG [980]

G.kre .T.TT.GC.T A.AG.TATGT G.CAC.CA.A ...------- ---------- ---------- ---------- ---------- ---------- ---------- ---------- ---------- ---------- ---------- [980]

C.azu ---------- ---------- ---------- ---------- ---------- ---------- ---------- ---------- ---------- ---------- ---------- ---------- ---------- ---------- [980]

V.var ---------- ---------- ---------- ---------- ---------- ---------- ---------- ---------- ---------- ---------- ---------- ---------- ---------- ---------- [1120]

V.mos ---------- ---------- ---------- ---------- ---------- ---------- ---------- ---------- ---------- ---------- ---------- ---------- ---------- ---------- [1120]

H.hip ---------- ---------- ---------- ---------- ---------- ---------- ---------- ---------- ---------- ---------- ---------- ---------- ---------- ---------- [1120]

H.ste TATCACAACC CCACCAGTTG TTTTAATAAT ACCACTTTTC AACCCACAAA TACCCCTGGC TTATCACAAC CCCACCAGTT GTTTTAATAA TACCACTTTT CAACCCACAA ATACCCCTGG CTTATCACAA CCCCACCAGT [1120]

R.hip AAAGATAAGC CCACCAGTTA TTTTAATAAT GGTACTTTTC AACCCTAAAA TACCCCTGAC CAAAGATAAG CCCACCAGTT ATTTTAATAA TGGTACTTTT CAACCCTAAA ATACCCCTGA CCAAAGATAA GCCCACCAGT [1120]

P.ste ---------- ---------- ---------- ---------- ---------- ---------- ---------- ---------- ---------- ---------- ---------- ---------- ---------- ---------- [1120]

P.oli AGACTTACCC TCAACCTTTC CCCCATTTTA ATGATT---- ---------- ---------- ------CAAC TTCTCTCACT CTTCATCTCA CCCTAACATG GGTTAGACTT ACCCTCAACC TTTCCCCCAT TTTAATGATT [1120]

S.max ATATAC---- ---------- ---------- ---------- ---------- ---------- ---------- ---------- ---------- ---------- ---------- ---------- ------AGTA TTATGTACAG [1120]

P.cor AACAACTA-- ---------- ---------- ---------- ---------- ---------- ---------- ---------- ---------- ---------- ---------- ---------- ------AAAG CCCTGGCCTG [1120]

G.kre ---------- ---------- ---------- ---------- ---------- ---------- ---------- ---------- ---------- ---------- ---------- ---------- ---------- ---------- [1120]

C.azu ---------- ---------- ---------- ---------- ---------- ---------- ---------- ---------- ---------- ---------- ---------- ---------- ---------- ---------- [1120]

V.var ---------- -----ACTTT TCAACCCTAA AATACCACTA GTAAACCACA AGCCCACCAG TTGTTTTAAT A-ATGGAACT TTTCAACCCT AAAATA-CCA CTAGTAAACC ACAAGCCCAC CAGTTGTTTT AATAATGGAA [1260]

V.mos ---------- -----..... .......... ....T..... A.C.....T. ...T...... .......... .-....T... .......... ......-T.. ...A.C.... .T....T... .......... ........T. [1260]

H.hip ---------- -----..... .......AC. ......C..G .CTT.T..T. .C........ .......... .-..ACC... .........A C.....-..C ..G.CTT.T. .T..C..... .......... ......ACC. [1260]

H.ste TGTTTTAATA ATACC..... .......AC. ......C..G .CTT.T.... .C........ .......... .-..ACC... .........A C.....-..C ..G.CTT.T. ....C..... .......... ......ACC. [1260]

R.hip TATTTTAATA ATGGT..... .......... ......C..G ACC..AG.T. .......... ..A....... .-....T... .......... ......-..C ..GACC..AG .T........ .....A.... ........T. [1260]

P.ste ---------- -----GA..C ........G. .GC.G.GG.G ACCC...... .A.TT....A ..A..G.... .-...TT.A. ...T...... ......-A.. GCG..CCG.. C..GA.TTT. ..-..A..AC ..C.-.ATTG [1260]

P.oli CAACTTCTCT CACTCTTCA. CTC....... C..GGGTTAG ACTT...CTC .A..TTT.CC CCA....... GAT.CA.CT. C.CTC..T.. TC.TCTCA.C ...AC.TGGG TT..A..A.. ..TGAACCCC TTATTGAAC. [1260]

S.max TATTATATAC AGTATTA.A. A..GTAT..T .TACAGTA.T A..T..AGT. TT---.TAT- ACAG.A.T.. .T.CA.T... A.AT-..AG. .TT...TA.. G..T..T.T- ...GTATT.T -.TACAG.A. T...T-AC.G [1260]

P.cor TAGCTTTTTA GGTATT..AC G-GG...... ..AGA.TAAC AA.C.GA.T. TTTG..TATT ACAA.A.T.C .-..ATT..A A.ATT..AA. .TT.C.-AT. T..CA.T.TT ....TATT.. -.-A.A..AC ....T.AC.. [1260]

G.kre ---------- ---------- ---------- ---------- ---------- ---------- ---------- ---------- ---------- ---------- ---------- ---------- ---------- ---------- [1260]

C.azu ---------- ---------- ---------- ---------- ---------- ---------- ---------- ---------- ---------- ---------- ---------- ---------- ---------- ---------- [1260]

V.var CTTTTCA-AC CCTAAAATAC CACTAGTAAA CC-ACAAGCC CACCAGTTGT TTTAATA-AT GGAACTTTTC AACCCTAAAA TA-CCACTAG TAAACCACAA GCCCACCAGT TGTTTTAATA ATGGAACTTT TCA-ACCCTA [1400]

V.mos .......-.. .........T .....A.C.. ..-.T....T .......... .......-.. ..T....... .......... ..-T.....A .C.....T.. ..T....... .......... ....T..... ...-...... [1400]

H.hip .......-.. ..AC...... .C..G.CTT. T.-GT..C.. .......... .......-.. ACC....... .....AC... ..-..C..G. CTT.T.GT.. C......... .......... ..ACC..... ...-....AC [1400]

H.ste .......-.. ..AC...... .C..G.CTT. T.-....C.. .......... .......-.. ACC....... .....AC... ..-..C..G. CTT.T..... C......... .......... ..ACC..... ...-....AC [1400]

R.hip .......-.. .......... .C..GACC.. AG-.T..... ........A. .......-.. ..T....... .......... ..-..C..GA CC..AG.T.. .......... .A........ ....T..... ...-...... [1400]

P.ste A..C...-.. ...G..GC.G .GG.GACCC. ..-....A.T T....A..A. .G.....-.. .TT.A....T .......... ..-A..GCG. .CCG..C..G A.TTT...-. .A..AC..C. -.ATTGA..C ...-.....G [1400]

P.oli AACCCTGCTA .T.TCCC..A AGTC.AA.T. TGGCT.TTT. ..T..T..CA GAC.CCCTCC CAC.TCCCCA CCT...GC.T G.CA..AA.. CT..AG.AC. CAAA.A.CAA CAG.A...G. -.ATTCAACA AG.G..A.AC [1400]

S.max TA..AT.T.. AG..TT...T -..AGTATT. TAT...GTAT T.TAT-ACAG .A.T...T.C A.T.T.A.AT -..AG..TT. ..TA..G..T ..T.T-...G TATT.T-.TA CAG.A.T... T-AC.GTA.. AT.T..AG.. [1400]

P.cor TA..A..-.T AT..C....T T..A.TATT. .A-.T.TTA. A.TATTACAA .A.T.C.-.. ATT..AA.AT T..AA..TT. C.-AT.T..C A.T.TT.... TATT..-.-A .A..AC.... T.AC..TA.. A..-.TAT.. [1400]

G.kre ---------- ---------- ---------- ---------- ---------- ---------- ---------- ---------- ---------- ---------- ---------- ---------- ---------- ---------- [1400]

C.azu ---------- ---------- ---------- ---------- ---------- ---------- ---------- ---------- ---------- ---------- ---------- ---------- ---------- ---------- [1400]

V.var AAATACCACT AGTAAACC-A CAAGCCCACC A-GTTGTTTT AATAATGGAA CTTTTCAACC CTAAAATA-C CACTAGTAAA CCACAAGCCC ACCAGTTGTT TTAATAATGG AACTTTTCA- ACCCTAAAAT AC--CACTAG [1540]

V.mos .....T.... .A.C....-. T....T.... .-........ ........T. .......... ........-T .....A.C.. ...T....T. .......... .......... T........- .......... .T--.....A [1540]

H.hip .......C.. G.CTT.T.-G T..C...... .-........ ......ACC. .......... .AC.....-. .C..G.CTT. T.GT..C... .......... ........AC C........- ....AC.... ..--.C..G. [1540]

H.ste .......C.. G.CTT.T.-. ...C...... .-........ ......ACC. .......... .AC.....-. .C..G.CTT. T.....C... .......... ........AC C........- ....AC.... ..--.C..G. [1540]

R.hip .......C.. GACC..AG-. T......... .-...A.... ........T. .......... ........-. .C..GACC.. AG.T...... .......A.. .......... T........- .......... ..--.C..GA [1540]

P.ste ..GC.G.GG. GACCC...-. ...A.TT... .-A..A..G. .......TT. A....T.... ........-A ..GCG..CC. -....CC... C..CCGCAC. CC..CT..C. CC.CC....- TGTTC..... ..--T.T..T [1540]

P.oli .CC.T..C.A CCCCTTA.A. GG.CTG..TG .CAA...G.C ...------- ---------- ---------- ---------- ---------- ---------- ---------- ---------- ---------- ---------- [1540]

S.max TT...T-..A GTATT.TAT. ..GTATT.TA T-ACA..A.. .TAT.CA.T. T.A.AT-..A G..TT...TA ..G..T..T. T-...GTATT .T-.TACAG. A.T...T-AC .GTA..AT.T ..AG..TT.. .T-A..G..T [1540]

P.cor C....TT..A .TATT..A-. T.TTA.A.TA TTACAA.GC. ..A...ATT. .AA.ATT..A A..TT.C.-A T.T..CA.T. TT....TATT ..-.-A.A.. AC....T.AC ..TA..A..- .TAT..C... .TTA..A..T [1540]

G.kre ---------- ---------- ---------- ---------- ---------- ---------- ---------- ---------- ---------- ---------- ---------- ---------- ---------- ---------- [1540]

C.azu ---------- ---------- ---------- ---------- ---------- ---------- ---------- ---------- ---------- ---------- ---------- ---------- ---------- ---------- [1540]

V.var TAAA-CCACA AGCCCAC--C AGTTGTTTTA ATAATGGAAC TTTTCAACCC TAAGCCAACC AAAACCCTCC TACACGCCAT CA--TCTCCT CATGGTCAAA AAACTGGTGT TTGGATTATT TC-AAG---- CATCTCTTAC [1680]

V.mos .C..-...T. ...T...--. .......... .......T.. .......... ...AAT.T.A CT..T.AA.. ATA.GCT..C ..GT.G.TT. A..AA.GGT. CTTT.CAACC C.AA.A...C A.T..T---- ..A.CA.A.G [1680]

H.hip CTT.-T.GT. .C.....--. .......... .....ACC.. C......... AC.AAT.C.. CTGG.TTAT. GTA..C...C ..GT.G.TT. A..AA.ACC. CTTT.CAACC CACA.A..CC C.TGGC---- TTATCG.A.. [1680]

H.ste CTT.-T.... .C.....--. .......... .....ACC.. .......... AC.AAT.C.. CTGG.TTAT. ACA..C...C ..GT.G.TT. A..AA.ACC. CTTT.CAACC CACA.A..CC C.TGGC---- TTATCACA.. [1680]

R.hip CC..-AG.T. .......--. ....A..... .......T.. .......... ...AAT.C.. CTG...AAAG ATA.GC...C ..GT.A.TT. A..AA.GGT. CTTT.CAACC C.AA.A..CC C.TG.C---- ..AAGA.A.G [1680]

P.ste .T.G---.TT .TTT..---- ...AT..CC. .C.CCA---- -CCC....TT CGG.A----- --GGTGTAAA ACA.AA..TC A.GGAACTAC AC.AC.TGT. GGCA..AACC --A..C.CGC C.T..----- -.G.CAGC.. [1680]

P.oli ---------- ---------- ---------- ---------- ---------- ---------- ---------- ---------- ---------- ---------- ---------- ---------- ---------- ---------- [1680]

S.max ..T.TA..GT .TTAT.T-A. ...AT.A.AT .C.G.ATT.T A.-A..GTAT ..TAT--..A GT.TTATAT- ACAGTATT.. -.TACAGTA. T..AT-AC.G T.TTATA--- -.AC.G.... ATAT.CAGTA T.ATATAC.G [1680]

P.cor ..C.-AT.TT .CAAT.TTA. .A.AT.ACA- ...T.AC..T A..A...TAT ..CA---.TA TT.CAATATT ACA.TATT.C -.-A.A.TAC A..AT.AC.. T.TTACAATA ..AC.A.GC. AAA..TATCG T.ATAT...T [1680]

G.kre ---------- ---------- ---------- ---------- ---------- ---------- ---------- ---------- ---------- ---------- ---------- ---------- ---------- ---------- [1680]

C.azu ---------- ---------- ---------- ---------- ---------- ---------- ---------- ---------- ---------- ---------- ---------- ---------- ---------- ---------- [1680]

V.var ATTACTACTA CTACAAAAGA CAGTGACTTT GCCCTACACC AAAACATCTT TTAATTAAC- --AAGTCCTC TAATCCTGCT ACCAAGAACT AACCTTTTCC TACCTCAAAA AACCTCCCAT GACAGCTCAA AACTGAC--- [1820]

V.mos C.C..C.G.T G.TTT..TA. TG..ACT... ---.A..C.T ....T...AC .-...C...C AT...CT.A. C.G.TG.TT. .AT..----. GGTAC...T. A...CT.... T.T.A.TA.. --..A.CAT. .G..C..CAG [1820]

H.hip CCC..C.G.T G.TTT..TA. T.CCACT... ---.A..C.A C...T.C.CC .-GGC.T.TC GT..CC..A. C.G.TG.TT. .AT..----. .C.AC...T. A...CAC... T...C.TGGC --TTATCGT. .C.CC..CAG [1820]

H.ste CCC..C.G.T G.TTT..TA. T.CCACT... ---.A..C.A C...T.C.CC .-GGC.T.TC AC..CC..A. C.G.TG.TT. .AT..----. .C.AC...T. A...CAC... T...C.TGGC --TTATCAC. .C.CC..CAG [1820]

R.hip CCC..C.G.T A.TTT..TA. TG..ACT... ---.A..C.T ....T.C.CC .-G.CC..AG AT...C..A. C.G.TA.TT. .AT..----. GGTAC...T. A...CT.... T...C.TG.C --..AAGAT. .G.CC..CAG [1820]

P.ste CCC.A..G-- GG.GT..C.C .G.GATA... -----..CT- ---------- ---------- ---------- ---------- ---------- ---------- ---------- ---------- ---------- ---------- [1820]

P.oli ---------- ---------- ---------- ---------- ---------- ---------- ---------- ---------- ---------- ---------- ---------- ---------- ---------- ---------- [1820]

S.max TA.TA..TAC AGTAT..TAT ACAGT.T.A. A--------T .C.GT..TA. A..CAGT.TT AT.T-A.AGT ATTATA-TAC .GT.TT.TA. .CAG.A..AT ACAACT.G.. T..AA.TAGA --ATA.AACT .GAAT..--- [1820]

P.cor .AC....T.T A.CA...TAC TCAAATT.G. .G---TGC.. .GG.T..T.A GA.CACTTTA ATGTTGTAAT GTTGTAATG. TGT..TGT-. GTAA.G..TT A.TG.TTT.. TGTT-TTA.. --GTTT.A.T GTT.T.A--- [1820]

G.kre ---------- ---------- ---------- ---------- ---------- ---------- ---------- ---------- ---------- ---------- ---------- ---------- ---------- ---------- [1820]

C.azu ---------- ---------- ---------- ---------- ---------- ---------- ---------- ---------- ---------- ---------- ---------- ---------- ---------- ---------- [1820]

V.var --GTCTGAGT CAAAATATTT T--AGCC--- ---------- ---------- ---------- ---------- ---------- ---------- ---------- ---------- ---------- ---------- ---------- [1960]

V.mos TT..T.T.A. A.TGG..C.. .TC.A..CTA AAATATCACT AATCAACCAT AAGCTCACCA GTTGTTTTAA TAATGGTACT TTTCAACCCT AAGACAACCA AAACCTTCCT ACACACCATC ATCT--CCTC GTGGTCAGAA [1960]

H.hip TT..T.T.A. A.T.CC.C.. .TC.A..CAC AAATACCCCT GGCTTATCGT AACCCCACCA GTTGTTTTAA TAATACCACT TTTCAACCCA CAAATACCTA AAACCCACAA ACGCCGTATC ACCT-TCTTC ATGATCAAAA [1960]

H.ste TT..T.T.A. A.T.CC.C.. .TC.A..CAC AAATACCCCT GGCTTATCAC AACCCCACCA GTTGTTTTAA TAATACCACT TTTCAACCCA CAAATACCGC AAACCCACAA ACGCCCTGTC ACCT-TCTTC ATGATCAAAA [1960]

R.hip TTA.T.T.A. A.TGG..C.. .TC.A..CTA AAATACCCCT GACCAAAGAT AAGCCCACCA GTTATTTTAA TAATGGTACT TTTCAACCCT AAAATACCCC TGACCAAAGA TAAGCCCACC AGTTATTTTA ATAATGGTAC [1960]

P.ste ---------- ---------- ---------- ---------- ---------- ---------- ---------- ---------- ---------- ---------- ---------- ---------- ---------- ---------- [1960]

P.oli ---------- ---------- ---------- ---------- ---------- ---------- ---------- ---------- ---------- ---------- ---------- ---------- ---------- ---------- [1960]

S.max -AACTA..A. AC..C..GAA .AC.A.TAGA ATACGCAC-- ---------- ---------- ---------- ---------- ---------- ---------- ---------- ---------- ---------- ---------- [1960]

P.cor -T..T.T.A. GTTTTA..G. .TT.ATGTTT TAATGTTTTA ATGTTTTAAT GTTTTAATGT TTTAATGTTT TAATGTTTTA ATGTTTTAAT GTTTTAATGT TTTAATGTTT TAATGTTTTA ATGTTTTAAT GTTTTAGCTT [1960]

G.kre ---------- ---------- ---------- ---------- ---------- ---------- ---------- ---------- ---------- ---------- ---------- ---------- ---------- ---------- [1960]

C.azu ---------- ---------- ---------- ---------- ---------- ---------- ---------- ---------- ---------- ---------- ---------- ---------- ---------- ---------- [1960]

V.var ---------- ---------- ---------- ---------- ---------- ---------- ---------- ---------- ---------- ---------- ---------- ---------- ---------- ---------- [2100]

V.mos AACTAGTGTT TGGATTATTT C-AAGCATCC CTTACACTAC CACTACCACA AAAGACAGTG ACTTTACCCT ACACCAAAAC ATCTTTTAAT TAACAAGTCC TCTAATCCTA CGAGGACTAA CCTTTTCTTA CCTCAGAAAG [2100]

H.hip AACTACTGTT TAGATTATTT C-AAGTATTC CTTTCACCAC CC-TAACTAC GAAAGGCATA AAACAGAATC CAAGAAAACT GCT--CCATC CAAAA----- CTATCTTTTT TAACCGATAA GCCCTTAAGT CCCCCTGAGA [2100]

H.ste AACTGCTGTT TAGATTATTT C-AAGTATTC CTTTCACCAC CC-TAACTAC GGAAGGCACA AAACAAAATC CAAGAAAACT GCT--CCATT CAAA------ TACCTTTTTT TAACCGATAG ACCCTTAAAT CCTCCTGAAA [2100]

R.hip TTTTCAACCC TAAAATACCC CTGACCAAAG ATAAGCCCAC CAGTTATTTT AATAATGGTA CTTTTCAACC CTAAAATACC CCTGACCAAA GATAAGCCCA CCAGTTATTT TAATAATGGT ACTTTTCAAC CCTAAAATGA [2100]

P.ste ---------- ---------- ---------- ---------- ---------- ---------- ---------- ---------- ---------- ---------- ---------- ---------- ---------- ---------- [2100]

P.oli ---------- ---------- ---------- ---------- ---------- ---------- ---------- ---------- ---------- ---------- ---------- ---------- ---------- ---------- [2100]

S.max ---------- ---------- ---------- ---------- ---------- ---------- ---------- ---------- ---------- ---------- ---------- ---------- ---------- ---------- [2100]

P.cor ---------- ---------- ---------- ---------- ---------- ---------- ---------- ---------- ---------- ---------- ---------- ---------- ---------- ---------- [2100]

G.kre ---------- ---------- ---------- ---------- ---------- ---------- ---------- ---------- ---------- ---------- ---------- ---------- ---------- ---------- [2100]

C.azu ---------- ---------- ---------- ---------- ---------- ---------- ---------- ---------- ---------- ---------- ---------- ---------- ---------- ---------- [2100]

V.var ---------- ---------- ---------- ---------- ---------- ---------- ---------- ---------- ---------- ---------- ---------- ---------- ---------- ---------- [2240]

V.mos CCTCCTATGA GAGCTCAAGA CTAGCGCCTG GGTCAGTTAA AATATTTAAC C--------- ---------- ---------- ---------- ---------- ---------- ---------- ---------- ---------- [2240]

H.hip ACCAGCCTTT TACCGCCCCA AAGGCTAAA- CACAAACAAA CTACCACCTC TAGGTGTGTT TAGAAAATTT ACT------- ---------- ---------- ---------- ---------- ---------- ---------- [2240]

H.ste ACCAGCCTTT TAACGCCCCA AAGGCTAAA- CACAAACAAA GTACCACCTC TAGATGTGGC TAGAAAATTT ACT------- ---------- ---------- ---------- ---------- ---------- ---------- [2240]

R.hip CTGGGACCTG TAACGCCGTG TCATCTTCTT CATGATCAAA AAACTACTGT TTAGATTATT TCAAGTATTT CTGGCATCAT CCCAACTACG AAGGGCACAG AACAAAAACC GAGAAGACTT TGCTCTCCAT TCAAAAACTA [2240]

P.ste ---------- ---------- ---------- ---------- ---------- ---------- ---------- ---------- ---------- ---------- ---------- ---------- ---------- ---------- [2240]

P.oli ---------- ---------- ---------- ---------- ---------- ---------- ---------- ---------- ---------- ---------- ---------- ---------- ---------- ---------- [2240]

S.max ---------- ---------- ---------- ---------- ---------- ---------- ---------- ---------- ---------- ---------- ---------- ---------- ---------- ---------- [2240]

P.cor ---------- ---------- ---------- ---------- ---------- ---------- ---------- ---------- ---------- ---------- ---------- ---------- ---------- ---------- [2240]

G.kre ---------- ---------- ---------- ---------- ---------- ---------- ---------- ---------- ---------- ---------- ---------- ---------- ---------- ---------- [2240]

C.azu ---------- ---------- ---------- ---------- ---------- ---------- ---------- ---------- ---------- ---------- ---------- ---------- ---------- ---------- [2240]

V.var ---------- ---------- ---------- ---------- ---------- ---------- ---------- ---------- ---------- ------ [2336]

V.mos ---------- ---------- ---------- ---------- ---------- ---------- ---------- ---------- ---------- ------ [2336]

H.hip ---------- ---------- ---------- ---------- ---------- ---------- ---------- ---------- ---------- ------ [2336]

H.ste ---------- ---------- ---------- ---------- ---------- ---------- ---------- ---------- ---------- ------ [2336]

R.hip TCTTTTTTTA ACCGACAAGC CCTTAAACCA ACCTTCTATT ATCCTAAAAC TTGACAAGAC TAGGGAAACA CCTTTTGGTG TAACAGATAT TTAACT [2336]

P.ste ---------- ---------- ---------- ---------- ---------- ---------- ---------- ---------- ---------- ------ [2336]

P.oli ---------- ---------- ---------- ---------- ---------- ---------- ---------- ---------- ---------- ------ [2336]

S.max ---------- ---------- ---------- ---------- ---------- ---------- ---------- ---------- ---------- ------ [2336]

P.cor ---------- ---------- ---------- ---------- ---------- ---------- ---------- ---------- ---------- ------ [2336]

G.kre ---------- ---------- ---------- ---------- ---------- ---------- ---------- ---------- ---------- ------ [2336]

C.azu ---------- ---------- ---------- ---------- ---------- ---------- ---------- ---------- ---------- ------ [2336]
